# Semibulk RNA-seq analysis as a convenient method for measuring gene expression statuses in a local cellular environment

**DOI:** 10.1038/s41598-022-19391-2

**Published:** 2022-09-12

**Authors:** Kyoko Muto, Issei Tsuchiya, Soo Hyeon Kim, Satoi Nagasawa, Mariko Takishita, Koichiro Tsugawa, Hiroaki Saito, Yusuke Komazaki, Toru Torii, Teruo Fujii, Yutaka Suzuki, Ayako Suzuki, Masahide Seki

**Affiliations:** 1 Department of Computational Biology and Medical Sciences, Graduate School of Frontier Sciences, The University of Tokyo, 5-1-5 Kashiwanoha, Kashiwa, Chiba 277-8561 Japan; 2 Institute of Industrial Science, The University of Tokyo, 4-6-1 Komaba, Meguro-ku, Tokyo, 153-8505 Japan; 3 Division of Breast and Endocrine Surgery, Department of Surgery, St. Marianna University School of Medicine, 2-16-1 Sugao, Miyamae-ku, Kawasaki, 216-8511 Japan; 4 Human Augmentation Research Center, National Institute of Advanced Industrial Science and Technology (AIST), Social Innovation Bldg., 6-2-3 Kashiwanoha, Kashiwa, Chiba 277-0802 Japan; 5 Department of Human and Engineered Environmental Studies, Graduate School of Frontier Sciences, The University of Tokyo, 5-1-5 Kashiwanoha, Kashiwa, Chiba 277-8561 Japan

**Keywords:** Gene expression analysis, Genome-wide analysis of gene expression, Cancer microenvironment, Next-generation sequencing, RNA sequencing

## Abstract

When biologically interpretation of the data obtained from the single-cell RNA sequencing (scRNA-seq) analysis is attempted, additional information on the location of the single cells, behavior of the surrounding cells, and the microenvironment they generate, would be very important. We developed an inexpensive, high throughput application while preserving spatial organization, named “semibulk RNA-seq” (sbRNA-seq). We utilized a microfluidic device specifically designed for the experiments to encapsulate both a barcoded bead and a cell aggregate (a semibulk) into a single droplet. Using sbRNA-seq, we firstly analyzed mouse kidney specimens. In the mouse model, we could associate the pathological information with the gene expression information. We validated the results using spatial transcriptome analysis and found them highly consistent. When we applied the sbRNA-seq analysis to the human breast cancer specimens, we identified spatial interactions between a particular population of immune cells and that of cancer-associated fibroblast cells, which were not precisely represented solely by the single-cell analysis. Semibulk analysis may provide a convenient and versatile method, compared to a standard spatial transcriptome sequencing platform, to associate spatial information with transcriptome information.

## Introduction

In the past decades, with the spread of next-generation sequencers, RNA sequencing has been used to measure gene expression profiles or identify transcripts. Especially, the single-cell RNA sequencing (scRNA-seq) has revolutionized the ability to identify and characterize diverse cell types and subpopulations. The currently most frequently used scRNA-seq methods employed the droplet method to separate the single cells^1,2^. Each cell is confined in a small droplet, in which the cell is lysed, and its mRNAs are independently barcoded. Several commercial platforms, such as Chromium of 10 × Genomics, were launched. Indeed, large-scale analyses have been conducted for various biological subjects. An international project, Human Cell Atlas, has also been launched to construct a comprehensive catalog of single cells, which are estimated 50–100 trillion in a given human body^3,4^. For cataloging mouse cells, Mouse Cell Atlas has also been launched^5–7^.

Despite its power, the first generation scRNA-seq did not give information for the surrounding context of the single cells. The droplet method inevitably required the cell dissociation, resulting in the loss of original tissue coordinates of the cell. However, the location of the single cells and the behavior of the surrounding cells is important to define the microenvironments, which are distinct depending on the cells even within a particular tissue. When cancer cells are exposed to hypoxia, they alter their metabolism to produce lactic acid^8^. The lactic acid microenvironment created by cancer cells induces and reprograms fibroblasts and macrophages around cancer cells^9–11^. These non-tumor cells secrete cytokines, chemokines, small RNAs, and metabolites that support the persistent survival of cancer cells^12^. For example, cancer-induced reprogrammed fibroblasts (CAFs) produce CXCL12, which contributes to cancer progression by further mobilizing bone marrow-derived cells in cancer tissue and promoting angiogenesis associated with cancer growth^13^. In this way, cancer cells adjust the surrounding microenvironment for their survival and create a metabolic symbiosis among multiple cells. Those changes further define the behavior of the cancer biology after that and, eventually, determine the clinical outcomes of the patients. Therefore, the full understanding of spatial information is essential for dissecting tissue-level systems biology.

Several methods have been proposed, collectively called spatial transcriptome (ST) analysis to map the spatial distributions of the cells in tissues^14–16^. The most representative platform is Visium system of 10 × Genomics^14,17^. Fresh frozen tissues have been used as starting material for Visium. Recently, Visium FFPE was released from 10 × Genomics, which employs split-probe ligation on mRNA instead of reverse transcription using polyA tail to detect fragmented mRNA^18^. This approach is expected to further expand the application of the VISIUM analyses. Indeed, these spatial approaches unravel the spatial information to some extent. Nonetheless, these methods are expensive, require high technical skills, and therefore difficult for many scientists to carry out. Furthermore, only a given planar of the sample can be analyzed with these methodologies, which sometimes do not reflect the actual RNA expression in the three-dimensional manner. Despite various advantages, it is still difficult to conduct ST analysis at clinical sites because of high cost, pressing time, or lack of technical skills.

On the other hand, the way to interpret the data from conventional bulk RNA-seq analyses has been substantially changed. With the increasing amount of the reference single-cell data, now it is possible, at least to some extent, to dissect the gene expression profile of the bulk tissue as the combination of the reference gene expression of the single cells that compose the tissue. Several computational methods, called deconvolution^19–25^, have been developed for this purpose, and its performance is rapidly improved.

This study developed an intermediate method between the droplet method and the conventional bulk RNA-seq method. In this method, a limited number of the cells are confined in a single droplet, called “semi” bulk cells. The multiple cell types in this semibulk co-exist with biological significance due to their close physical proximity. The above example is the relationship between cancer cells, CAFs, and tumor-associated macrophages. The semibulk is a cellular unit that preserves multispecies cell–cell interactions that can be collected without breaking the physical distance that reflects this biological significance. By taking advantage of the high throughput, which is one of the advantages of the method developed in this study, thousands of semibulk cells were analyzed simultaneously. We further dissected the obtained information of each of the semibulk cells to its composing single cells by the deconvolution. Since semibulk cells retain the local information of the cells, we expected to obtain the semi-spatial information from the analysis. The semibulk cells are an easier sample for some clinical settings. The semibulk is the very cell mass observed in cytodiagnosis, which has long been practiced in clinical practice. For example, the biopsy samples harvested by fine-needle absorptions could be directly used for this analysis. We first developed a method for generating the droplet having an appropriate size for this purpose. Using the developed system, we subjected the mouse kidney and compared the data with Visium ST data. We also conducted the semibulk analysis using clinical breast cancer specimens for the proof of concept.

## Results

### Workflow of sbRNA-seq analysis

**Figure 1:**
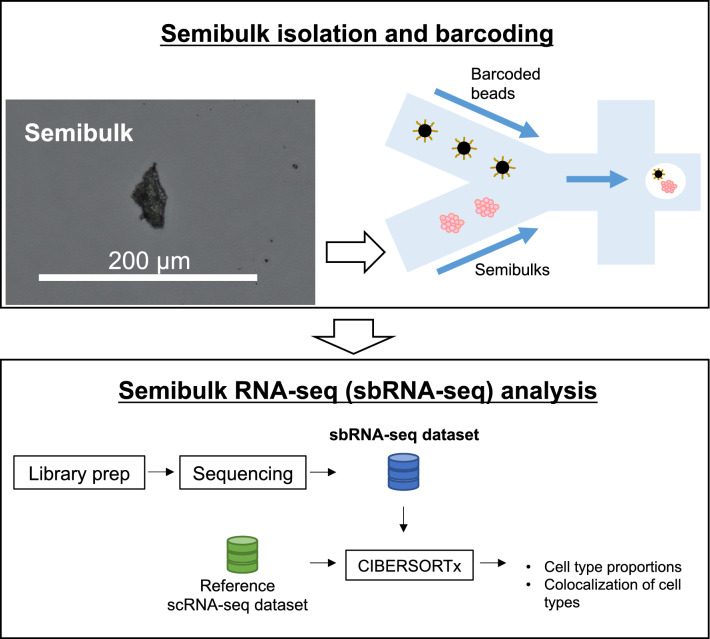
A workflow of sbRNA-seq. Overall schematic representation. Cell aggregates from tissues are isolated and mixed with a barcoded bead and reaction solution within a droplet. In the droplet, reverse transcription is conducted to generate barcoded cDNA. Library preparation and sequencing are performed to generate sbRNA-seq data. After sequencing, deconvolution analysis is conducted using CIBERSORTx^25^ to estimate the cell-type proportion of each semibulk using scRNA-seq data as a reference.

We constructed a novel device and method to capture thousands of “semibulks”, which are tissue pieces with 100–200 cells and perform RNA-seq analysis of barcoded semibulks (Fig. 1). Semibulk RNA-seq (sbRNA-seq) was designed with the following steps. (1) Semibulk suspension was acquired from respective tissues. We expect that mechanical dissociation as conducted here should be relatively less damaging, as it does not require incubation time, which may allow RNA degradation. Moreover, cryopreservation causes cellular damage, which may result in RNA degradation. To evaluate the cellular damage induced by mechanical dissociation, we performed trypan blue staining of semibulks prepared from murine kidney (Supplementary Fig. 1). Except for a small portion of cells and tissue gaps, almost no cells were stained, showing that they are alive. We avoided the enzymatic treatment since it may also weaken the cell adhesion within semibulks. Harvested tissues were minced into small pieces (semibulks). An average size of semibulk ranged approximately 40–200 µm. Because the minimum channel diameter of the 10 × chromium is ~ 50 µm (Supplementary Fig. 2a), the channel would be clogged with semibulks of 50–200 µm. To overcome this issue, we fabricated a PDMS device in which the channel diameter is larger than semibulks. We set the width and height of the device into 250 µm for generation of droplets with ≥ 250 µm in diameter, which is sufficient to encapsulate both a barcoded bead and a semibulk (Supplementary Fig. 2b). Once, the device is prepared, it can be re-used many times; (2) Each semibulk was encapsulated with a distinctly barcoded microparticle (bead) using the above-developed nanoliter-scale droplet generator; (3) Semibulks were lysed after having been isolated in the droplets; (4) mRNAs were released from the semibulk cells and, then, were captured on its companion bead. The captured mRNA was reverse transcribed, amplified, and converted to the sequencing templates by following the standard procedure for the scRNA-seq. Sequencing was conducted for more than 1000 and 100 semibulks per sample for murine kidney and human clinical specimens. The single semibulk gene expression information was obtained according to the barcode information. (5) Cell-type components of each semibulks were predicted by deconvolution analysis.

### Microfluidic device for encapsulating semibulks with barcoded beads

**Figure 2:**
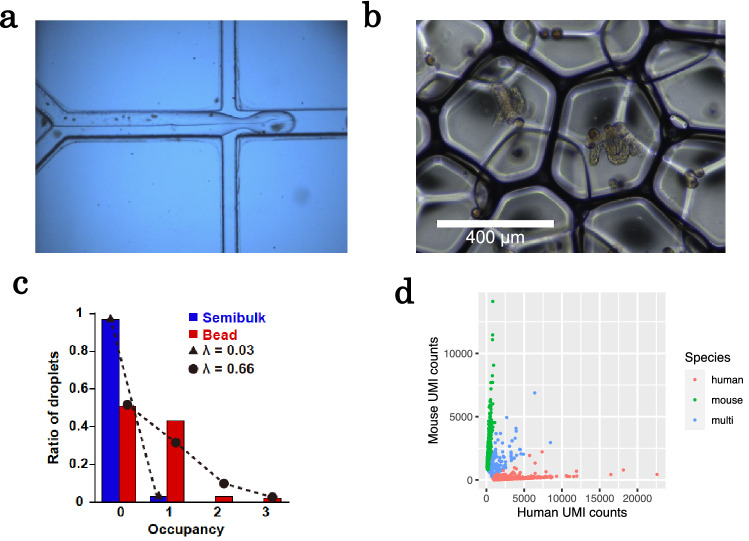
Encapsulation of semibulks with barcoded beads and cross-species validation. (**a**) A picture of microfluidic device for microdroplet generation. (**b**) A picture of microdroplets. (**c**) The distribution of ratio of droplets (containing from zero to three beads or semibulks) fitted with the Poisson distribution. The fitted expected values λ for semibulks and beads were 0.03 and 0.66, respectively. (**d**) UMI counts, aligned to human and mouse genomes, of the data from the cell mixture. 1451 (47.6%), 1416 (46.4%), and 184 cells (6.0%) were judged as human, mouse cells, and multiplets of these cells, respectively.

We designed a microfluidic device to encapsulate large semibulks with barcoded beads. This device quickly flows two aqueous solutions across an oil channel to form more than 7000 nanoliter-sized droplets per minute. One flow contains the barcoded beads suspended in a lysis buffer; the other flow contains semibulks (Fig. 2a). The two flows were mixed in the microfluidic channel before entering the cross-junction used for the droplet generation, which prevents close contamination between semibulks.

We demonstrated pairing of single bead and single semibulk by water in oil droplets with barcoded beads and semibulks suspended in an aqueous solution. The encapsulation rate of beads or semibulks follows the Poisson distribution (1)^26^, where expected value λ represents the average number of beads (or semibulks) per droplet, *k* represents the number of microbeads (or semibulks) encapsulated in a droplet, and *p* represents the probability of the droplets which contains *k*-beads (or semibulks).

1 $$p( {k,\lambda } ) = \frac{{\lambda^{k} e^{ - \lambda } }}{k!}$$

To prevent encapsulation of multiple beads with a semibulk or multiple semibulks with a bead, we decreased the concentration of beads or semibulks so that λ was less than 1 to get a small probability of the droplets which contain multiple microbeads or semibulks. After droplet generation, images of droplets were taken using microscopy (Fig. 2b). Then, we counted the number of droplets which contain zero, one, two, three beads or semibulks, and the distribution of ratio of droplets were fitted with the Poisson distribution (Fig. 2c). The fitted λ for semibulks and beads were 0.03 and 0.66, respectively. The ratio of droplets that contain single semibulk and bead was 0.024. Since the encapsulation of beads is independent of semibulks, the paring efficiency could be calculated by multiplying the λ of semibulks and beads (0.020), which is quite well matched with the experimentally obtained ratio of droplets which contains single semibulk and bead. This result indicates that the encapsulation of beads and semibulks are follows the Poisson distribution and one can estimate the pairing efficiency from the λ of semibulk and bead.

### Cross-species validation using a mixture of human and mouse cell lines

To evaluate multiplet rate of the custom device, we conducted cross-species validation. For cross-species validation analysis, we used a 1:1 mixture of mouse (NIH3T3) and human (HEK293) cell lines at 10 cells/µL to the custom microfluidics device for sbRNA-seq (Supplementary Table 1). The obtained reads were aligned to the respective reference genomes. A total of 3051 cells were detected, whose UMI counts were > 1000. To distinguish single-cell data derived from multiplet of mouse and human cells, we counted cells with > 25% of UMI-normalized reads aligned to both genomes. The multiplet rate of human and mouse was 6.0% (Fig. 2d). This number is reasonable compared with the 10 × Chromium system, where the mixture rate is estimated at 7.6% and 3.9% at the captured cell counts of 10,000 and 5000, respectively. The semibulk concentration in this study was ≤ 4 semibulks/µL. Therefore, we concluded that the tissue multiplet rate should be < 6.0%.

### Application of the sbRNA-seq for mouse kidney

**Figure 3:**
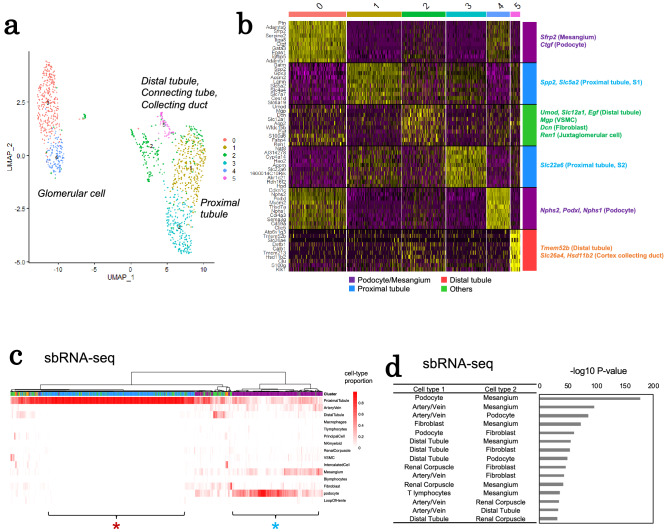
Evaluation of sbRNA-seq using mouse tissues. (**a**) A UMAP plot of 1158 sbRNA-seq datasets generated by Seurat^24^. Six clusters are shown in each color. (**b**) A heatmap of differentially expressed genes in each cluster of (**a**). Representative cell-type markers manually represented in the margin. The heatmap was prepared using Seurat. (**c**) Cell-type proportions of mouse kidney sbRNA-seq inferred by CIBERSORTx^25^. The upper color keys show semibulk classifications manually annotated from cell-type markers in (**b**). The heatmap of cellular-type proportions was prepared using pheatmap of R. (**d**) Probabilities of co-occurrence of two cell types in sbRNA-seq datasets. The hypothesis test of independence of cell-type pairs was conducted. The horizontal bar plot shows the − log_10_ P-value of the top 15 pairs of cell types with the lowest P-values.

Using the developed device for semibulk generation, we conducted sbRNA-seq using a mouse kidney tissue. A kidney should be an organ model with an arrayed local unit structure, nephron. We obtained 1158 sbRNA-seq datasets in which each of the semibulks represents more than 1000 expressed genes (Table 1). Clustering analysis of sbRNA-seq appeared as shown in Fig. 3a on the UMAP plot. Overall, six clusters appeared. We extracted characteristic gene expression to annotate each cluster, as often employed in scRNA-seq analysis (Fig. 3b). By particularly focusing on typical marker genes (as shown on the vertical axis; Fig. 3b), we were able to identify the major components of each cluster as indicated (on the horizontal axis; Fig. 3b). For example, for clusters 0 and 4, specific expression of the representative genes for podocyte/mesangial cells, such as *Sfrp2*, *Ctgf*, *Podxl*, *Nphs1*, and *Nphs2,* were observed^27–29^. The clusters 1 and 3 showed the highest expression of the marker genes for proximal tubule, such as *Spp2*, *Slc5a2*, and *Slc22a6*. The cluster 2 appeared as a mixture of aggregates of various cell types in the cortex and outer medulla (for the gene expression levels of the particular marker genes, see Supplementary Fig. 3). Based on these results, we concluded that sbRNA-seq analysis should represent a mixture of the cellular components with varying cellular compositions as expected.

**Table 1:** Summary of mouse kidney sbRNA-seq.

	Mouse kidney
Total number of reads	430,582,143
Number of reads after filtering by UMI-tools	102,353,183
Number of uniquely mapped reads	67,829,111
% Uniquely mapped reads	66.3
Number of semibulks	1158
Mean reads per semibulk	371,833
Total UMI counts of semibulks	5,772,865
Median UMI counts per semibulk	3353
Median genes per semibulk	1614
% Mitochondrial UMI counts	0.2

To further examine whether the sbRNA-seq should be useful for obtaining local cell-type compositions, we inspected the sbRNA-seq data to determine whether the positional information of each semibulk can be precisely extracted. To evaluate this issue, we attempted to dissect each sbRNA-seq gene expression information to its component cells by referring the single-cell gene expression information of each cell type. For the reference single-cell information, previously published single-cell gene expression catalogs were employed^27^. For deconvolution of cellular components using the component single-cell gene expression patterns, CIBERSORTx^25^, which is the most frequently used program for this purpose^30–35^, was used. The deconvolution result of mouse kidney semibulks appeared as shown in Fig. 3c. We compared the results of a deconvolution analysis using Seurat, SPOTlight, and RCTD^22–24^ (Supplementary Figs. 4 and 5), and the results suggest that CIBERSORTx is a reasonable bioinformatics tool, at least for our purpose. For example, the semibulk indicated by the red asterisk mostly consisted of proximal tubular cells. On the other hand, the semibulk indicated by the blue asterisk are mainly composed of podocytes. The concordant result with the clustering was shown. Also note that in several semibulks, components of blood cells were also represented.

To evaluate the precise representation of spatial information, we examined whether the anatomically expected cellular proximity information should be correctly represented. Based on the estimated cellular component ratio in each semibulk, we calculated the possibility of colocalization of two cell types in the same semibulk. As a result, podocytes, mesangial cells (mesangium), and vascular cells (artery/vein) were in the same semibulk with high possibilities (Fig. 3d). These cell types are components of glomerulus^36^. For blood filtration in the kidney, small arteries bring bloods into glomeruli capillaries close to podocytes. The result of sbRNA-seq deconvolution is consistent with anatomical structures^27,37^. Other associations for which the significant spatial proximity was observed are shown in Fig. 3d. Most of the indicated associations were consistent with anatomical information.

### Comparison with spatial transcriptomic data of mouse kidney

**Figure 4:**
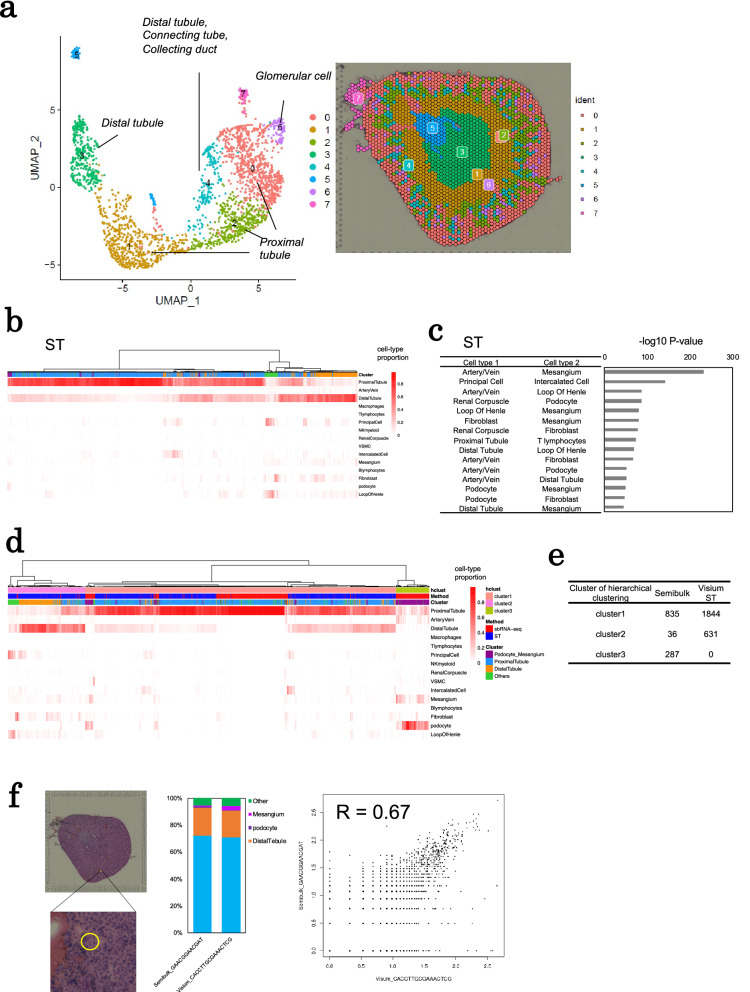
Comparison with spatial transcriptomic data of mouse tissues. (**a**) A UMAP plot of 2477 Visium spots (left). Seven clusters are shown in each color. Distribution of these clusters on the tissue section was also shown (right). (**b**) Cell-type proportions of each spot of mouse kidney Visium ST datasets inferred by CIBERSORTx^25^. The heatmap shows cellular-type proportions. The upper color keys show spot classifications manually annotated from cell-type markers in Supplementary Fig. 7. (**c**) Probabilities of co-occurrence of two cell types in ST datasets. The hypothesis test of independence of cell-type pairs was conducted. The horizontal bar plot shows the − log_10_ P-value of the top 15 pairs of cell types with the lowest P-values. (**d**) Hierarchical clustering of deconvolution results from sbRNA-seq and ST of mouse kidney. The heatmap shows cellular-type proportions. The color keys at the top of the figure show the clusters obtained from pheatmap and cutree functions of R, whether the data is from sbRNA-seq or ST, and manually annotated cell types in Fig. 3b and Supplementary Fig. 7, respectively. (**e**) Number of semibulks or spots from sbRNA-seq or ST belonging to each cluster obtained by hierarchical clustering. (**f**) Comparison of expression patterns among multiple platforms, sbRNA-seq and ST. Hematoxylin–eosin image of ST, the cell fraction for the semibulk and Visium spot, and the scattering plot of gene expression between them. The Pearson correlation coefficient is shown in the graph.

For validation of the performance of sbRNA-seq analysis, we performed representative ST analysis, Visium, for mouse kidney using the same mouse strain with sbRNA-seq (Supplementary Table 2) and compared the result of sbRNA-seq with ST data. In previous studies, the spatial patterns of cell–cell interactions in the kidney were detected by Visium^38,39^. Expression levels of 2477 spots were detected by ST. Given the uniformity of UMI counts^40^ and the number of cells manually counted, our Visium planar should have a relatively flat cellular density across the observed region (Supplementary Fig. 6). From clustering analysis of ST, seven clusters separated on the tissue section appeared (Fig. 4a). We annotated each cluster using the typical marker genes also used for annotation of sbRNA-seq data (Supplementary Fig. 7). For example, for clusters 6, specific expression of the representative genes for podocyte/mesangial cells, such as *Sfrp2*, *Podxl*, *Nphs1*, and *Nphs2,* were observed^27–29^ (Supplementary Fig. 8). Clusters 0, 1, and 2 showed the highest expression of marker genes for proximal tubule, such as *Spp2*, *Slc5a2*, *Slc22a6*, and *Slc22a7*. Clusters 3 and 4 showed the highest expression of marker genes for distal tubules, such as *Slc12a1*, *Egf*, and *Umod*. We also performed deconvolution of cellular components in Visium spot (Fig. 4b). We visualized cellular components obtained by deconvolution on the tissue section (Supplementary Fig. 9). Most cell types showed a distribution consistent with the anatomical information^27^. For example, the proximal tubule was in the cortex. On the other hand, the loop of henle is in the inner medulla and the distal tubule in the cortex and outer medulla. We also calculated the possibility of colocalization of two cell types in the same Visium spot (Fig. 4c), and detected colocalization of glomerular cells (podocytes, mesangium, and artery/vein) from ST data.

To evaluate the similarity of the fraction of the cellular components between sbRNA-seq and Visium, we performed hierarchical clustering of the composition data, which was estimated from the sbRNA-seq and Visium data. From the hierarchical clustering analysis, we found that 75% of the sbRNA-seq datasets were clustered with ST dataset according to similarities of cell-type compositions (Fig. 4d,e). For example, one semibulk composed of the proximal tubule and distal tubule showed a similar expression pattern with a Visium spot located in the cortex (r = 0.67, Fig. 4f). Another one semibulk composed from proximal tubule and podocytes showed a similar expression pattern with a Visium spot (r = 0.61, Supplementary Fig. 10). This spot located in cortex and expressed *Nphs1* which is a podocyte marker.

Although the data showing the similar expression patterns and cell-type components with ST analysis could be obtained also from sbRNA-seq analysis (Fig. 4f and Supplementary Fig. 10), we also noticed that the data points corresponding to glomerular cells in ST were less represented than in sbRNA-seq (Fig. 4d and Supplementary Fig. 11). From the hierarchical clustering analysis, we identified three clusters (Fig. 4e): Cluster 1 primarily contained proximal tubular cells, Cluster 2 mainly contained distal tubular cells, and Cluster 3 contained glomerular cells, such as podocytes and mesangial cells. While Clusters 1 and 2 were composed of semibulk and Visium, Cluster 3 was composed of only semibulks. Visium collectively measures the sum of the expression levels of spatially neighboring cells, which are located within the two-dimensional distance of a given spot (55 μm). In other words, a dot may represent the gene expression of the cells located in the neighborhood, even if they do not have any physical connection. On the other hand, sbRNA-seq measures that of physically connecting cells, because otherwise, the cells should be separated during sample preparation. Therefore, Visium should detect the glomerulus with distal tubular cells, which are located in the neighborhood (< 55 μm)^37^ and are included in Cluster 2. Because these cells do not have strong cellular connection, this interaction may not be represented by sbRNA-seq. In addition, in a real kidney, the volume of the cortex is larger than that of the medulla^41^. We consider that the difference in the representation between semibulks and Visium may also be accounted for the fact that sbRNA-seq analyzes minced tissues uniformly for the whole kidney, whereas Visium only analyzes the spots on the designated planar, including a wider medullar space compared with the whole kidney (Supplementary Fig. 11). Although larger amounts of starting tissue material are needed for sbRNA-seq than ST, sbRNA-seq would be more convenient than ST in detecting the interaction of glomerular cells. These results indicated that expression patterns of sbRNA-seq and estimated cellular compositions were reasonable for measuring transcriptome statuses distinct to local space of tissues.

Collectively, these results showed that sbRNA-seq should give useful information to represent local components in a reasonably accurate manner.

### sbRNA-seq analysis of human breast cancer specimens

**Figure 5:**
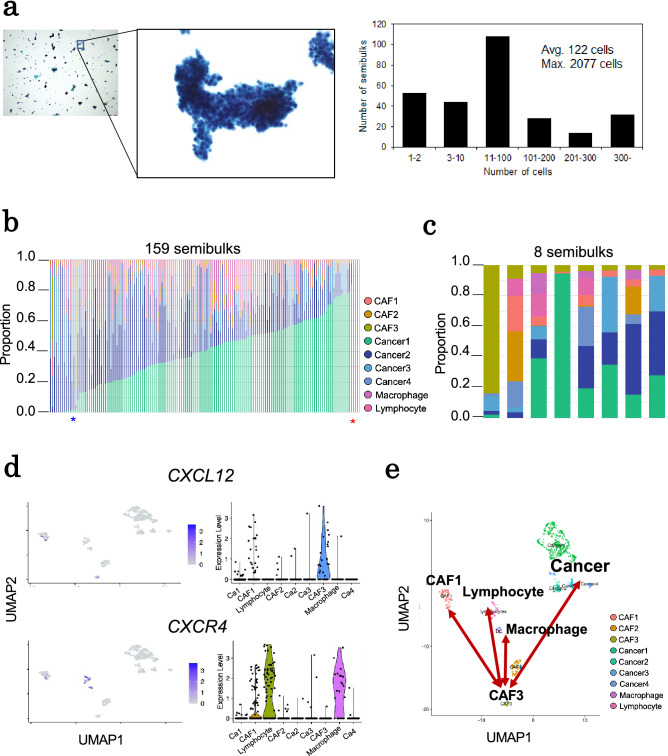
Application of sbRNA-seq for breast cancer specimen (Case A). (**a**) Papanicolaou-stained image of a pseudo-semibulk created from Case A (left panel). In addition to cancer cells, lymphocytes, fibroblasts, and other cells form aggregates. The distribution of estimated cells in each semibulk is represented in the right panel. One semibulk contains an average of 122 cells. (**b**) Cell-type proportions of sbRNA-seq inferred by CIBERSORTx. The color key of cell types is shown in margin. (**c**) Cell-type proportions of eight semibulks containing > 3% CAF3 are shown. Macrophages, CAF1 and lymphocytes present in the same semibulks are in physical proximity to CAF3. (**d**) Using single-cell data of Case A, expression levels of *CXCL12* and *CXCR4* genes are represented for each cell type. *CXCL12* is mainly expressed in CAF3, while *CXCR4* is mainly expressed in macrophages and lymphocytes. (**e**) The illustration of association between each cell type of Case A on the UMAP plot of scRNA-seq.

To apply the thereby developed sbRNA-seq method, we used two human breast cancer clinical specimens (a pathological image; Supplementary Fig. 12). These specimens were obtained from two patients. Case A is a typical case of invasive cancer (Supplementary Fig. 12a). In this case, the cancer cells spread to all area of the specimen. On the other hand, Case B is a case of relatively early-stage cancer, in which most of the cancer cells were still confined within milk ducts (ductal carcinoma in situ, DCIS) (Supplementary Fig. 12b). Further details of the patients’ background are shown in Table 2. In this study, even though these samples were surgical materials, we treated them as a mock sample of biopsy samples and fine-needle absorption samples, for which both usual scRNA-seq and ST analysis are frequently impossible. An example view of the Papanicolaou-stained sbRNA-seq sample is shown in Fig. 5a. We examined and found that an average of 122 cells were included in one semibulk. For the reference gene expression information for the deconvolution, scRNA-seq analysis was also performed for the same material (Supplementary Fig. 13). This time, we collected the scRNA-seq data by ourselves on the Chromium platform. However, even this part would be bypassed, when the public scRNA-seq database of breast cancers is enriched in the near future. Summary of the sbRNA-seq and the scRNA-seq datasets is shown in Table 3 and Supplementary Table 3.

**Table 2:** Clinical information of two breast cancer cases.

	Case A	Case B
Type	Invasive ductal carcinoma	Encapsulated papillary carcinoma
Age	77	43
ER (IHC)	Positive	Positive
PgR (IHC)	Positive	Positive
HER2 (IHC)	Negative	Negative

*ER* estrogen receptor, *PgR* progesterone receptor, *HER2* human epidermal growth factor receptor 2, *IHC* immunohistochemistry.

**Table 3:** Summary of sbRNA-seq of breast cancer specimens.

	Case A	Case B
Total number of reads	304,336,141	317,199,357
Number of reads after filtering by UMI-tools	115,660,595	90,715,267
Number of uniquely mapped reads	59,150,516	60,855,970
% Uniquely mapped reads	51.1	67.1
Number of semibulks	159	116
Mean reads per semibulk	1,914,064	2,734,477
Total UMI counts	148,552	318,382
Median UMI counts per semibulk	556	1745
Median genes per semibulk	328	879.5
% Mitochondrial UMI counts	5.9	3.7

Firstly, we obtained and analyzed a total of 159 semibulks in the Case A specimen. Since we could not use abundant clinical specimens in this study, fewer semibulks than for mouse were captured and used for the analysis. In addition, maybe due to the fact that it took longer collection time for the clinical specimens after the surgery, RNA quality was lower, as also indicated by the higher mitochondrial UMI counts than in mouse samples. We thought that the lower number of detected genes in clinical samples should be also derived from the quality of the starting materials (Table 3). The results of the initial clustering analysis appeared as shown in Supplementary Fig. 14a. Relevant clusters were not observed for sbRNA-seq data representing the relatively uniform representation of the sbRNA-seq within the specimen. On the other hand, in the scRNA-seq data, clusters were clearly separated to according to the component cell types. Using the obtained datasets, we conducted the annotation of the spatial information simultaneously with gene expression imputations for each semibulk. For the imputation, CIBERSORTx analysis for sbRNA-seq data uses the scRNA-seq data as a reference similar to the mouse kidney. scRNA-seq data indicated roughly four clusters of cancer cells (Cancer1–4), three clusters for cancer-associated fibroblast cells (CAF1–3), macrophage groups, and lymphocytes. The results of the imputation analysis of sbRNA-seq indicated that each semibulk should consist of a combination of these cell types (Fig. 5b). For example, one sbRNA-seq, indicated by the red asterisk in Fig. 5b, mostly consisted of cancer cells, while another semibulk (blue asterisk) mostly consisted of CAFs. In fact, various combinations of the cell types at varying mixing rates were observed.

We further inspected the sbRNA-seq regarding the spatial representation. The cell-type proportion of eight semibulks containing > 3% CAF3 are shown in Fig. 5c. We found that a population of cancer-associated fibroblast (CAF3 as of scRNA-seq cluster) are significantly associated with several groups of immune cells, particularly that of macrophages (p = 0.047). When we examined the gene expressions of cytokines and their receptors on the scRNA-seq data which may represent possible interactions, we found that a gene expression of an inflammatory-related chemokine gene, *CXCL12*, was remarkably induced in CAF3 (Fig. 5d). Such induction was not observed for the other CAF clusters, CAF1 and CAF2. On the immune cell side, the gene expression of*CXCR4*, which is the receptor of CXCL12, was particularly induced in macrophages and lymphocytes. These results indicated that CAF3 may have recruited the macrophages and lymphocytes via the CXCL12-CXCR4 interaction (Fig. 5e). Such CAF3 has been reported to be activated by cancer to produce CXCL12^13^, but little is known about the biological characteristics of CAF-activating cancers. Therefore, to understand the biological characteristics of Cancer4 co-occurring CAF3, we conducted Gene set enrichment analysis with top 30 upregulated differentially expressed genes of the Cancer4 using Metascape^42^ (Supplementary Fig. 15a). Four key pathways associated with cancer hallmarks were affected in the cancer4. In particular, the key genes and pivotal pathways associated with G2/M DNA damage checkpoint was included. The G2/M DNA damage checkpoint regulation pathway has been reported^43^ the top-ranked pathways at the early stage of hepatocellular carcinoma (HCC), Cancer4 may be the cancer cell with features that prepare the microenvironment for invasion.

**Figure 6:**
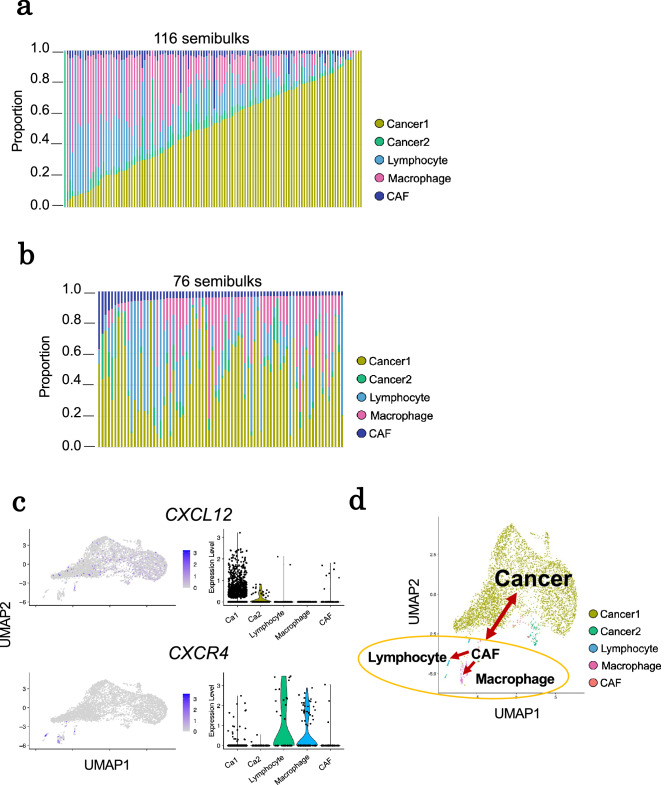
Application of sbRNA-seq for breast cancer specimen (Case B). (**a**) Cell-type proportions of 116 sbRNA-seq inferred by CIBERSORTx. The color key of cell types is shown in the margin. (**b**) Cell-type proportions of 76 semibulks containing > 3% CAF3 were shown. (**c**) Expression patterns of *CXCL12* and *CXCR4* in five cell-type clusters of scRNA-seq. (**d**) The potential association between each cell type shown on the UMAP plot of scRNA-seq data in Case B.

We also examined the results of the sbRNA-seq analysis in Case B specimen as to whether a similar phenomenon should be represented. A total of 116 semibulks were obtained and subjected to a similar analysis (Fig. 6a and Supplementary Fig. 14b). In Case B, the cellular components and other features were significantly different from Case A, in particular, the total number of immune/stromal fractions was low, reflecting distinct states of the cancers between them. In this Case B specimen, we particularly focused on the possible interaction between CAFs and macrophages. We found that 76 semibulks included significant fractions of CAFs (> 3%) (Fig. 6b), and 67% (51 of 76) of them also contained macrophages (> 3%). We observed the similar induced gene expression of *CXCL12* and *CXCR4* in CAFs and macrophage clusters, respectively (Fig. 6c), which indicated that small fractions of CAF3-like CAFs of CaseA might also exist in the CAF population of Case B. Interestingly, *CXCL12* was also expressed in cancer cells (Cancer1 and Cancer2) and in particular, Cancer2 which was a fraction of proliferating tumor cells and was also enriched in the G2/M DNA damage checkpoint (Supplementary Fig. 15b) significantly co-exists with macrophages (p = 0.0053). These results indicate that similar macrophage recruitment may occur in this Case B, which was associated with the construction of invasive niche (Fig. 6d). Perhaps reflecting that the cancers and CAFs are less exposed to immune cells, a smaller population may have represented the population of the interacting CAFs and macrophages in this case.

### Improvement and evaluation of efficiency of sbRNA-seq

To improve the efficiency of the sbRNA-seq analysis, we have performed additional sequencing analysis for all the sbRNA-seq libraries. For the mouse kidney data, the median genes per semibulk increased from 1614 to 1700, and that of the captured semibulks increased from 1158 to 1495 (Table 1 and Supplementary Table 4). For the libraries of clinical samples, while the median number of genes per semibulk slightly decreased from 328–880 to 234–754, the number of the captured semibulks increased from 116–159 to 199–568 (Table 3 and Supplementary Table 5). These results suggested that even removed semibulks by quality filtering could be rescued by increasing sequence depth, though yet deeper sequencing may be needed to enrich the data content for each semibulk at least for the mouse kidney data (Supplementary Fig. 16).

To confirm the efficiency of the sbRNA-seq analysis, we conducted an experimental replicate of the sbRNA-seq of the mouse kidney by constructing a similar sbRNA-seq library (Supplementary Table 4). By applying the concentrated suspension (~ 4 semibulks/µL) to the droplet system, the number of captured semibulks increased to 3205. At a sequencing depth of ~ 1 billion reads for this library, a median of 1196 genes were represented per semibulk. Due to significantly lower mean reads per semibulk in the replicate (63% of the previous data), the median number of genes per cell (1196) is somewhat lower than in previous data (1700). The median genes per cells should increase with increasing sequence depth. To evaluate the batch effect between replicates, we performed UMAP clustering using Seurat with default settings (Supplementary Fig. 17). The replicates were clustered together showing minimum if any batch effects between replicates. This result showed high reproducibility of sbRNA-seq. Since the protocol of sbRNA-seq is based on the original Drop-seq^1^, the improvement of microbeads and reaction conditions used in this method would further increase gene detection efficiency.

## Discussion

This paper described the development of novel technology, sbRNA-seq. By sbRNA-seq, we tried to provide a technically easier and more cost-effective platform to analyze the spatial organization of the gene expressions in tissues. Key features that distinguish it from the other platforms include a PDMS microfluidic device designed for semibulks which can be used to analyze several thousands of semibulks in a single experiment. The filtered samples can be directly applied to this system without enzyme, which reduces the damage to mRNAs.

In the first part, we introduced the fabrication method of microfluidic device. The lane width can be changed conveniently, so it can be modified to fit your own purpose. In the latter part, we validated and demonstrated the performance of the developed sbRNA-seq approach. For validation purposes, we generated the sbRNA-seq datasets of mouse kidneys. As shown in Fig. 3, we demonstrated that known intercellular interactions can be captured in the mouse kidney data, which could not be analyzed solely by scRNA-seq analysis. Therefore, we found that this analysis could obtain information about the local cellular interactions or local cellular neighborhood in a reasonably precise manner. Finally, we applied the developed sbRNA-seq technology to analyze two human breast cancer patients (Figs. 5 and 6). As a result, we found that the CXCL12–CXCR4 axis is associated with the local cellular interaction between immune cells and cancer-associated fibroblasts. We believe further accumulation of such a biological insight should have substantial clinical relevance, especially when the corresponding patient is treated with cancer immunotherapy. We also believe sbRNA-seq analysis can be applied to a wide variety of biological contexts.

Because sbRNA-seq is a unique method, there is no other equivalent method to conduct the similar analysis. Therefore, strictly precise comparison of our data with that of other methods was not possible. Some practical features were compared as shown in Supplementary Table 6. We admit that our system for sbRNA-seq may be still preliminary, especially regarding its efficiency for data collection, and should more space for further improvements as partly demonstrated in Supplementary Tables 4 and 5. There are even still remaining parts in the protocol susceptible of further improvement, such as bead size and its number as suggested by the reviewer, which may further increase the performance.

Although sbRNA-seq should be applicable regardless of tissue type, we think that sbRNA-seq would be particularly useful, for example, for tumor cell analyses, since they are clustered within malignant ascites and pleural effusion of cancer patients. Because those cell clusters are composed of tumor cells and other stromal cells, the analysis of those cell clusters should represent the nature of tumor cells in their residing microenvironments^44^. Moreover, in some tumor types, such as pancreatic cancer and lung small cell carcinoma, cancer cells are sparsely and three-dimensionally distributed and not operable in most cases. Therefore, it is difficult to obtain detailed information using the current ST, which requires use of surgery materials. In addition, cases of inoperable, relapsing, or metastasizing cancers are unique targets, as far as biopsy samples are available via FNA. Moreover, sbRNA-seq is also useful for analyses of cell species not suitable for droplet-based scRNA-seq, such as multinuclear giant cells (for example, osteoclasts and myocytes), whose diameter is > 50 µm, and neuronal cells, which are sensitive to single-cell dissociation.

As represented in this study, sbRNA-seq can be employed to analyze multiple species and tissues or organs. This method performs well with heterogeneous tissues and can be used with other types of diseases as well. Many researchers have tried to define a comprehensive cell atlas in humans and other species. Recent genome field trends have generated large-scale single-cell datasets. With the speed of data generation, new techniques to combine these datasets and dissect other types of cell states such as protein and chromatin levels have been established. Our way of interpreting biological mechanisms is shifting from simply conducting single-cell analysis to combining these single-cell data with other forms of data. What is now wanted is to connect this scattered information into consistent data to understand tissue-level systems biology.

## Materials and methods

### Device fabrication

The microfluidic channel for droplet generation was fabricated by PDMS (Silopt 184; Dow Corning Toray, Co. Ltd.) using a standard replica molding process. Chromium patterns for microfluidic channel fabricated on the soda lime glass were outsourced. The mold master was fabricated directly on the photomask to make high aspect ratio structure for the PDMS molding. 5.4 g of negative photoresist was dropped onto the photomask and spin-coated at 300 rpm for 8 s and 500 rpm for 30 s. Subsequently, the photomask was baked with hot plate at 65 °C for 10 min and 100 °C for 80 min. Photomask was reversed to make the spin-coated side face down and placed in ultraviolet radiation machine. It was exposed until the total energy irradiation attained 1.5 W. Afterwards, photomask was baked with hot plate at 65 °C for 3 min and 100 °C for 10 min. The photomask was cooled and immersed into liquid developer (SU-8 developer). It was shaken with a shaker for 30 min until the area except the pattern was removed. It was then rinsed with isopropanol and ion exchange water. Base compound and curing agent of PDMS were mixed in 10 to 1 proportion and the mixture (100 g) was poured onto the mold. Then it was placed in a desiccator and defoamed with a compressor to remove air bubbles inside the PDMS or between the PDMS and mold. The mold master with PDMS was placed in an oven and baked for 150 min at 75 °C. Cured PDMS was cut into the size of the device, and access ports (1.5 mm in diameter) were opened. PDMS sheet and glass sheet were placed in Reacting Ion Etching and exposed by oxygen plasma at 1000 mTorr for 30 s. Both were aligned, brought into contact, and spontaneously bonded together without external pressure. Adhered PDMS microfluidic device was placed in an oven and baked for 180 min at 150 °C.

### Preparation of cell mixture

A human cell line, HEK293 (ATCC, CRL-1573), and mouse cell line NIH3T3 were cultivated in DMEM medium containing l-glutamine (Wako) supplemented with 10% FBS (Corning) and 1 × Antibiotic–Antimycotic (ThermoFisher Scientific). Cultivated cells were dissociated using 0.25% trypsin + EDTA (ThermoFisher Scientific). After washing by medium and D-PBS (–) (Wako), cells were suspended in PBS + 0.01% BSA (Sigma-Aldrich), and the cell suspension was filtered. Cell concentrations were measured using Countess IIFL (ThermoFisher Scientific). The same number of human and mouse cells were mixed, and the cell concentration was adjusted to 10 cells/µL with PBS-BSA.

### Ethical declaration and tissue preparation

All animal experiments were approved by the animal care and use committee of Graduate School of Frontier Sciences, the University of Tokyo (approval number: C-16-2) and was conducted in accordance with the guidelines and regulations for proper conduct of animal experiments of the University of Tokyo. This study was reported in accordance with ARRIVE guidelines. Healthy mice (ICR, female, 10 weeks, Japan SLC) were used for the experiments. After the mice were euthanized with isoflurane, murine kidneys were harvested. All procedures using human clinical samples were performed in accordance with Japanese ethical guidelines for medical and health research involving human subjects and human genome/gene analysis research. Human breast cancer tissues were collected from two women undergoing surgery for breast cancer with the appropriate informed consent at St. Marianna University Hospital, Japan (approval number: 2297-i103). Experimental procedures were also approved under the same approval number. Case A is derived from the same specimens as a tumor tissue of Case 8 used in our previous study^45^. We subjected whole tumor tissues to dissection of a few mm in thickness without excluding a specific area to prepare single cells and semibulks.

### Semibulk tissue disaggregation

The samples were washed with PBS and minced using a razor blade and scissors. Samples were placed in 500 µL PBS, passed through a cell strainer pluriStrainer 40 µm (pluriSelect Life Science), and washed with PBS to remove red blood cells and single cells. The bulks that remained on the cell strainer were passed through a pluriStrainer 200 µm (pluriSelect Life Science) again to remove huge bulks. When the volume of the solution was too large, the solutions were centrifuged for 5 min at 500×*g*, 4 °C, and supernatant was removed. Neither red blood cell lysis nor specific lysis using enzymes were performed on all samples. For dead cells and cell debris, we did not count them during sample preparation or could not estimate them from the data. 3 mL of solution was applied to sbRNA-seq library preparation.

### Library preparation and sequencing for sbRNA-seq

sbRNA-seq libraries were prepared following the protocol provided by McCarroll Lab (Drop-Seq Laboratory Protocol, version 3.1)^1^. This procedure included breakage of droplets, reverse transcription, exonuclease I treatment, PCR, purification of cDNA library, tagmentation of cDNA, and purification of tagmented library. Sequencing was performed on Illumina HiSeq 3000 or NovaSeq 6000.

### Library preparation and sequencing for Visium spatial transcriptome dataset

Mouse of the same strain (ICR strain; female; 10 weeks old) were used for both sbRNA-seq and Visium, but not the same individual mouse. The kidney was frozen by spray freezing with OCT compounds. A 10-µm cryosection was prepared by cryostat and put on the Visium Spatial Gene Expression Slide (Visium Spatial Gene Expression Slide Kit, 10 × Genomics). Fixation and H&E staining were conducted according to the Tissue Fixation and Staining Demonstrated Protocols provided from 10 × Genomics. H&E visualization was conducted using BZ-X800 (KEYENCE). Tissue permeabilization, cDNA amplification, and library preparation were performed using Visium Spatial Gene Expression Reagent Kits according to the manufacturer’s protocol. For tissue permeabilization, the incubation time was set to 18 min. Sequencing was performed using Illumina NovaSeq 6000.

### Single-cell isolation from breast cancer tissues

Human breast cancer tissues were obtained from surgical resection specimens. Samples were washed with PBS, mechanically disaggregated using razor blade, and digested in DMEM (Wako) containing 2 mg/mL collagenase Type P (Roche). Before library preparation, cellular debris and aggregates were filtered out using a 40 µm filter.

### Library preparation and computational analysis of scRNA-seq of breast cancer

The scRNA-seq libraries of breast cancer tissues were prepared following the 10 × Genomics Chromium Single Cell Gene Expression Solution protocol. Single-cell suspensions were counted using Countess (ThermoFisher Scientific) and adjusted to 300–400 cells/µL. Cells were loaded according to the protocol of the Chromium single cell 3′ kit to recover around 3000 cells. Version 3 chemistry was used. Sequencing was performed on an Illumina HiSeq 3000. scRNA-seq data was processed using Cell Ranger (version 3.1.0). The generated count matrices were further analyzed using Seurat v4.0^24^. Briefly, scRNA-seq datasets with less than 500 expressed genes or > 5% mitochondrial counts were discarded. We labeled major cell populations using expression patterns of cell-type markers as below; cancer cells: *EPCAM*, *ESR1*, *PGR*, *ERBB2*, *MKI67*, *KRT7*, *KRT8*, *KRT18*, and *KRT19*; cancer-associated fibroblasts: *POSTN*, *MMP11*, *COL1A1*, *COL3A1*, *COL5A1*, *COL5A2*, *COL6A3*, *ANGPT1*, *ANGPT2*, *THY1*, *SPARC*, *SPARCL1*, *VIM*, *CAV1*, *FN1*, *FAP*, *COL6A1*, and *COL6A2*; lymphocytes: *CD2*, *CD3D*, *CD3E*, *CD3G*, *CD4*, *CD8A*, *CD8B*, *FOXP3*, *CD19*, and *CD79*; macrophages: *CD163*, *CD14*, *CSF1R*, *CD33*, *CD68*, and *FTL*.

### Computational analysis of scRNA-seq from human and mouse cell mixture

From the total reads, reads with UMIs and cell barcodes were extracted using UMI-tools v1.1.1^46^. Extracted sequences were mapped against a reference genome combining mouse genome (mm10) and human genome (hg38) using STAR v2.6.1a^47^ and SAMtools v1.9^48^. UMI-normalized reads aligned to each genome were counted. Data for which > 25% of UMI-normalized reads were aligned to both genomes, was judged as a multiplet of human and mouse cells. Gene expression levels were calculated using featureCounts^49^.

### Computational analysis of sbRNA-seq from mouse kidney

From the total reads, the reads with UMIs and cell barcodes were extracted using UMI-tools v1.1.0^46^. Extracted sequences were mapped against mouse genome (mm10) using STAR v2.6.1a^47^ and SAMtools v1.9^48^. Expression levels were calculated using featureCounts^49^.

The generated count matrices were further analyzed using Seurat v4.0.3^24^. Briefly, datasets with less than 1000 expressed genes or > 25% mitochondrial counts were discarded. Then, dimensional reduction was conducted by principal component (PC) analysis. Two-dimensional visualization by UMAP and clustering analysis were performed using 10 PCs.

To analyze mouse kidney, the cell-type composition was estimated using CIBERSORTx v1.0^25^ with default parameters. Reference datasets were constructed using expression levels of each cell-type cluster from the mouse kidney scRNA-seq data previously reported^27^. Briefly, the count matrix under the accession number GSE129798 was downloaded from Gene Expression Omnibus. Clustering analysis was performed using Seurat v4.0.3^24^. The clusters of scRNA-seq data were classified to 15 cell types, including mesangium and podocyte in addition to the 13 cell types in the previous study^27^. To identify each cluster, cell-type marker genes in the study^27^ and the following genes were used; mesangium: *Sfrp2*; podocyte: *Nphs1/2*. Because the web version of CIBERSORTx could not process a large reference dataset for mouse kidney (31,265 cells), we used the local version of CIBERSORTx. Briefly, we first created a signature matrix with CIBERSORTx from the reference of the indicated scRNA-seq data. Cell-type fractions for each semibulk were then estimated by CIBERSORTx using the generated signature matrix.

Probabilities of co-occurrence of two cell types were evaluated by the chi-square test. Each semibulk was classified based on whether its percentage of a cell type was higher than that of the median (“present” or “absent”). For each cell-type pair, the number of semibulks was calculated in every four types (present/absent × present/absent), and P-values were estimated from the chi-square test of independence. The data were discarded if the existence ratio in the presence of the other cell type was lower than the overall ratio.

### Validation analysis using the Visium spatial transcriptome dataset

Visium fastq files were processed using SpaceRanger v1.2. The processed datasets were analyzed using Seurat v4.0 and CIBERSORTx v1.0 in the same way as the analysis of sbRNA-seq.

### Computational analysis of sbRNA-seq from breast cancer tissues

Sequences were processed and mapped to the human reference genome (hg38) similarly to mouse analysis. The generated count matrices were also analyzed using Seurat v4.0^24^. Briefly, sbRNA-seq datasets with < 200 expressed genes or > 25% mitochondrial counts were discarded for Case A. sbRNA-seq datasets with < 500 expressed genes or > 25% mitochondrial counts were discarded for Case B. Cell-type deconvolution was performed using the web version of CIBERSORTx v1.0 similarly to the mouse analysis. Reference datasets were constructed using expression levels of each cell-type cluster from the breast cancer scRNA-seq data obtained from the same cases. Probabilities of co-occurrence of two cell types were evaluated in a similar manner to mouse kidney analysis.

## Supplementary Information

Supplementary Figures.

Supplementary Information.

## Data Availability

All sbRNA-seq data from mouse tissues was deposited to DRA (https://www.ddbj.nig.ac.jp/dra/index-e.html) or GEA (https://www.ddbj.nig.ac.jp/gea/index-e.html) of DNA Data Bank of Japan (DDBJ) under the accession numbers, DRA014313 (sbRNA-seq for kidney), DRA014314 (scRNA-seq for cell mixture, additional sequencing of sbRNA-seq for kidney, and experimental replicate of sbRNA-seq for kidney), DRA013006 (Visium), and E-GEAD-462 (Visium). Sequencing data of clinical samples is available in the Japanese Genotype–Phenotype Archive (JGA, https://www.ddbj.nig.ac.jp/jga/index-e.html), which is hosted by the National Bioscience Database Center (NBDC) and DDBJ with the identifiers JGAS000387.
