# Supplementary material for: Semibulk RNA-seq analysis as a convenient method for measuring gene expression statuses in a local cellular environment
Source: Sci Rep. 2022 Sep 12;12:15309. doi: 10.1038/s41598-022-19391-2 (PMC9468030; doi:10.1038/s41598-022-19391-2)
Supplement: Supplementary file 1 — Supplementary Figures. [file 41598_2022_19391_MOESM1_ESM.pdf]

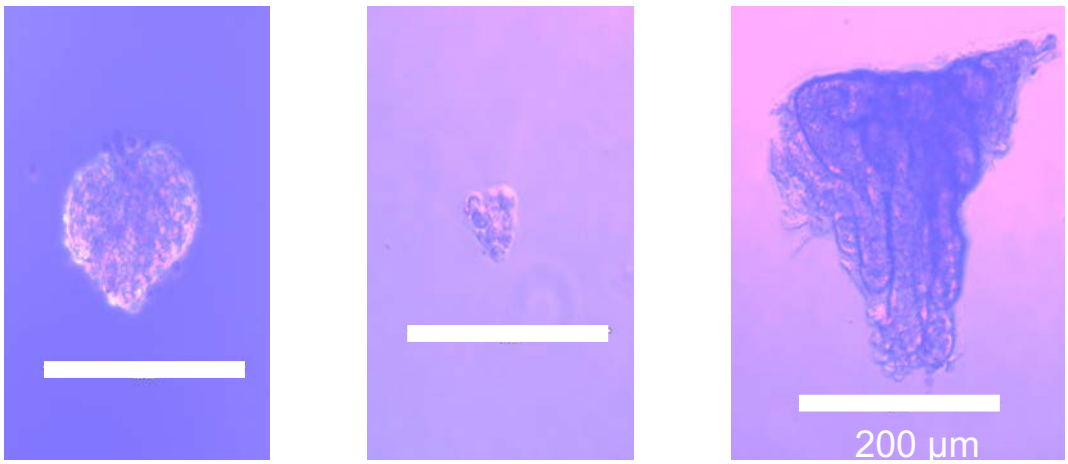

Supplementary Figure 1

a

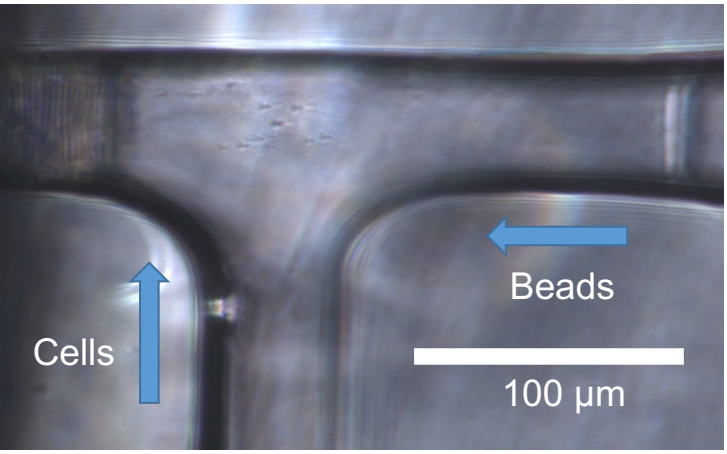

b

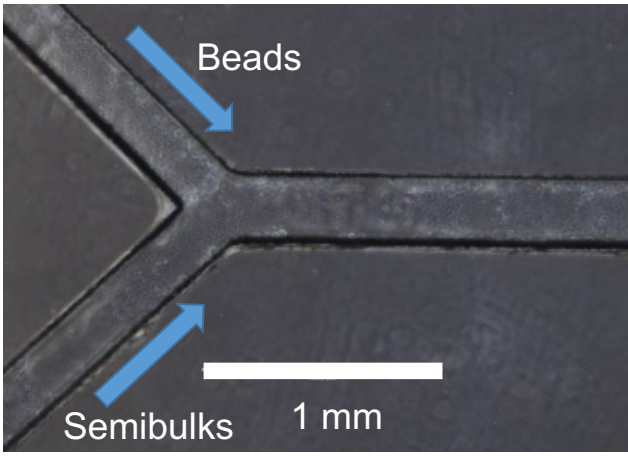

Supplementary Figure 2

Proximal tubule

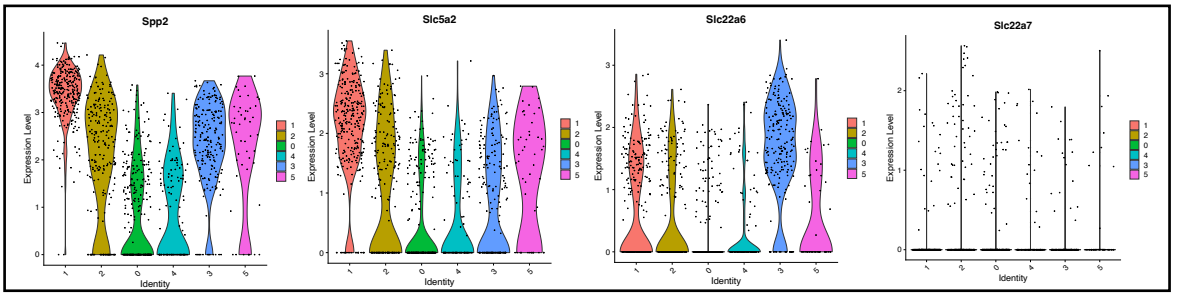

Distal tubule

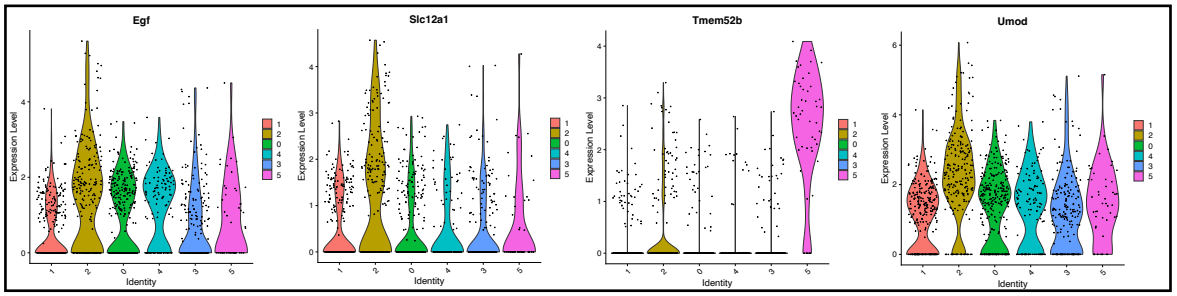

Podocyte

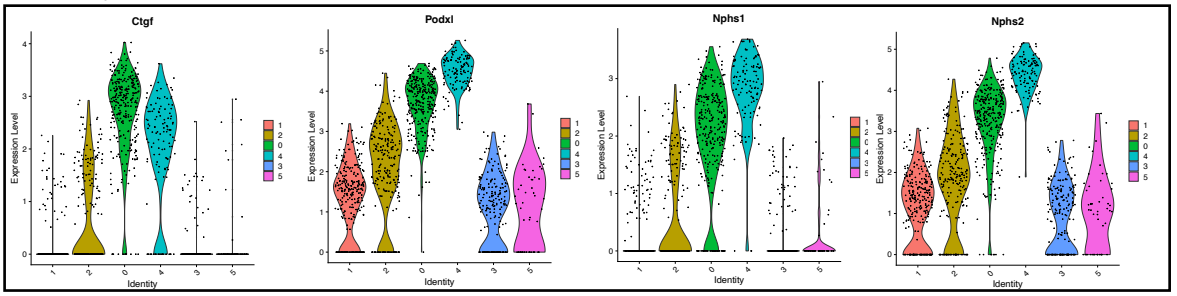

Juxtaglomerular cell

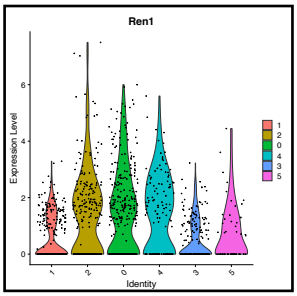

Mesangium

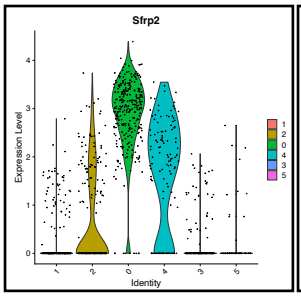

Fibroblast

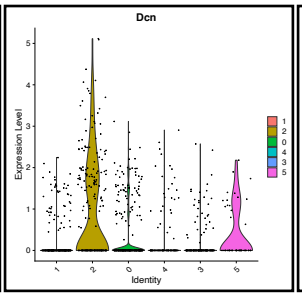

VSMC

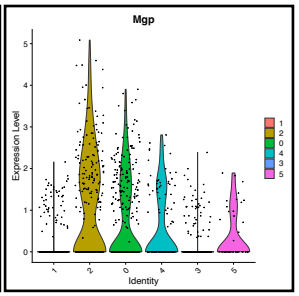

Collecting duct

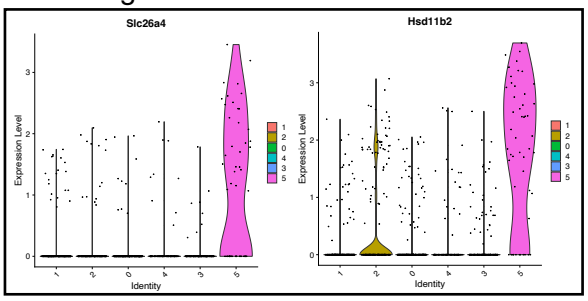

Supplementary Figure 3

Seurat

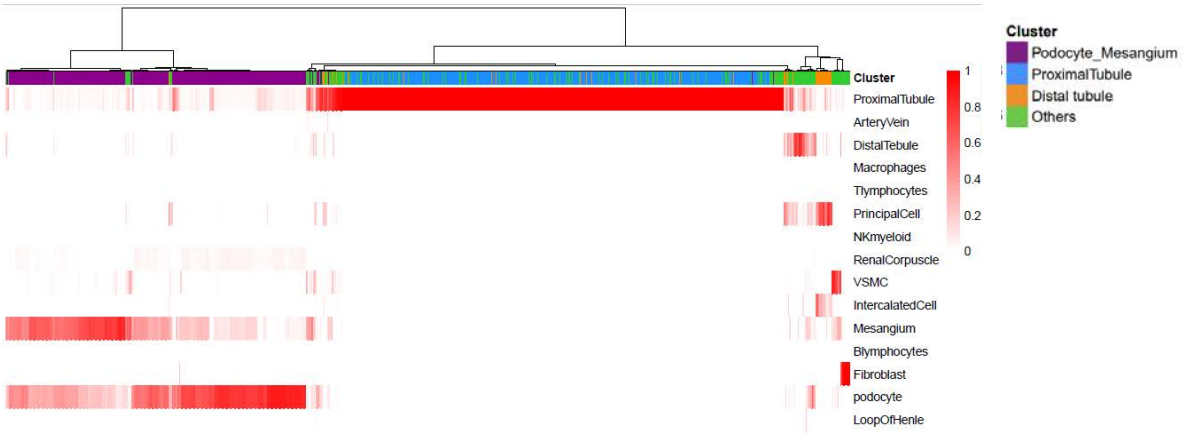

RCTD

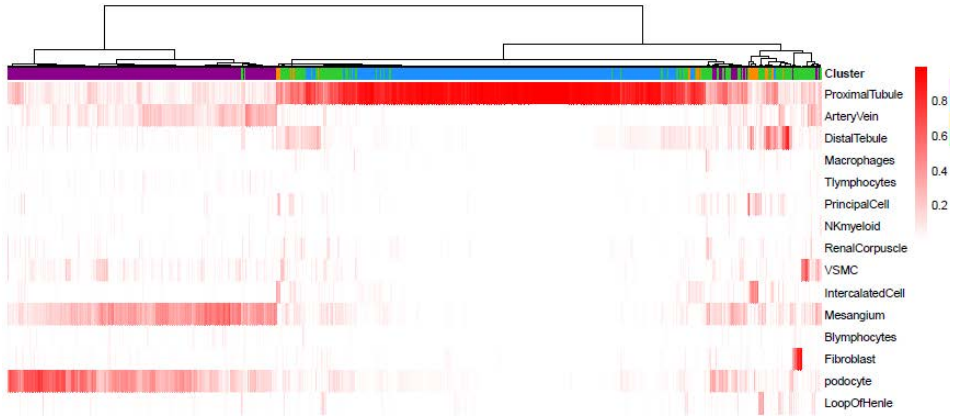

SPOTlight

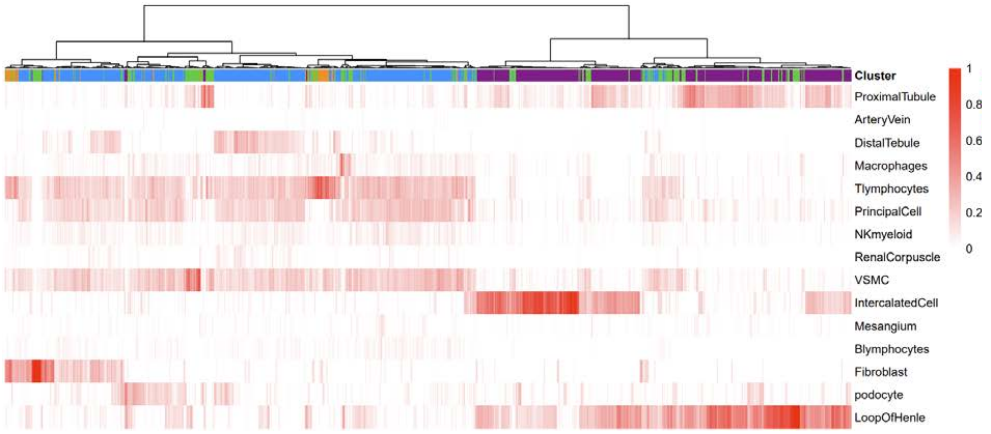

Supplementary Figure 4

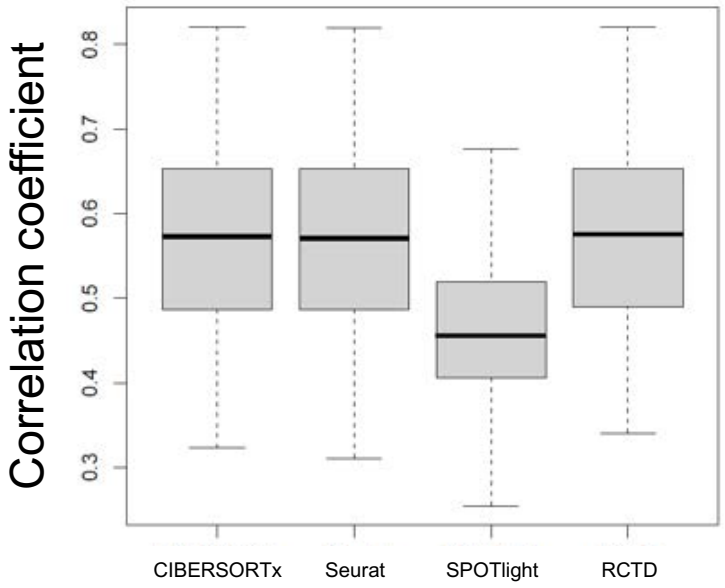

Supplementary Figure 5

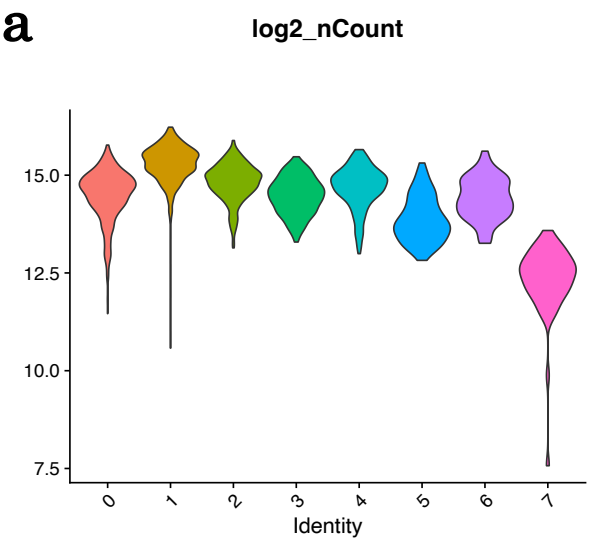

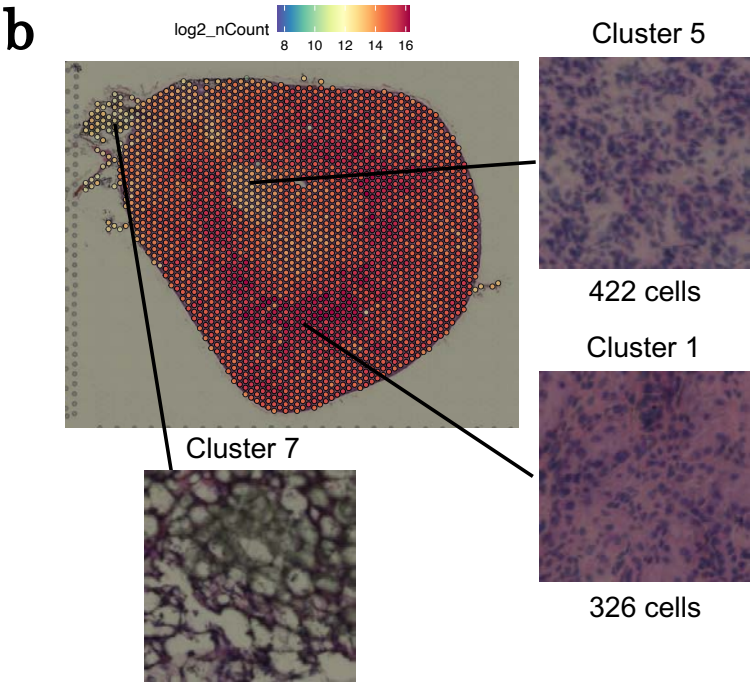

Supplementary Figure 6

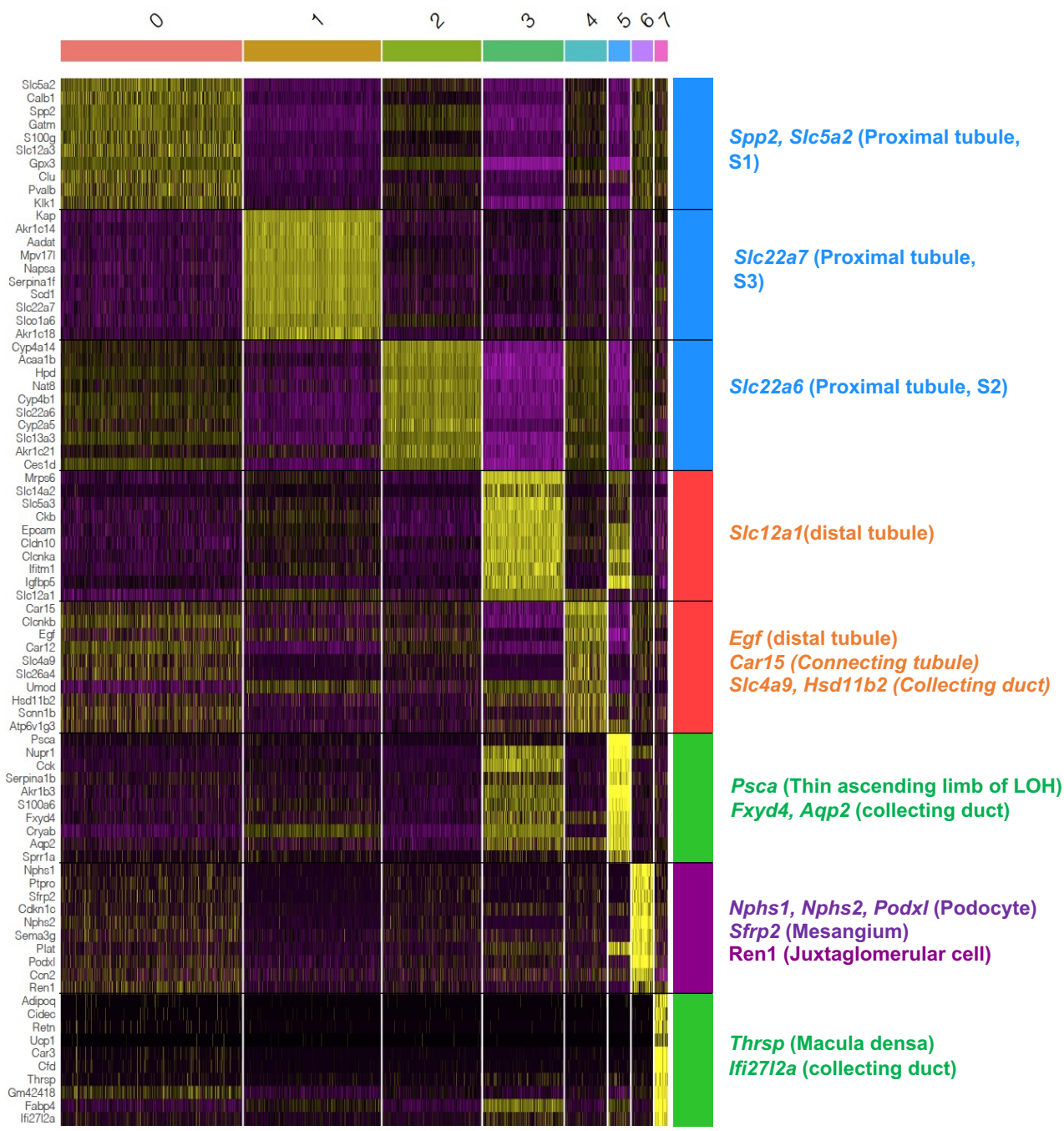

Supplementary Figure 7

Proximal tubule

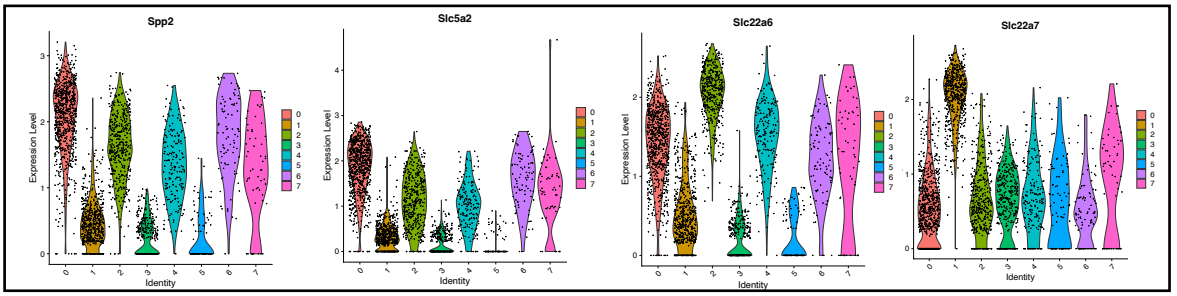

Distal tubule

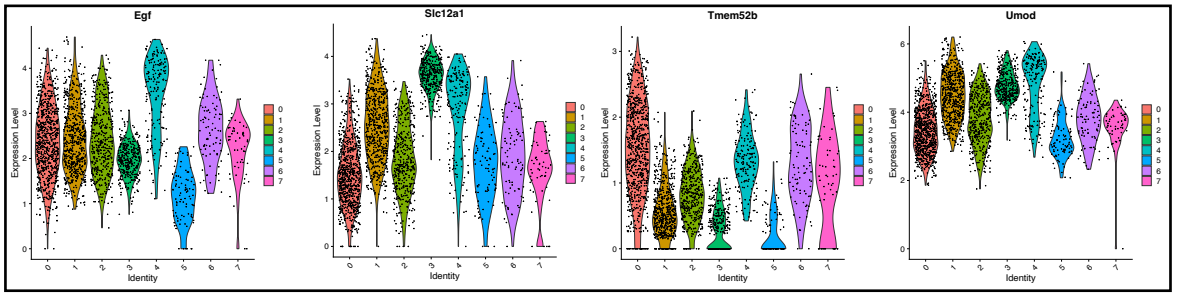

Podocyte

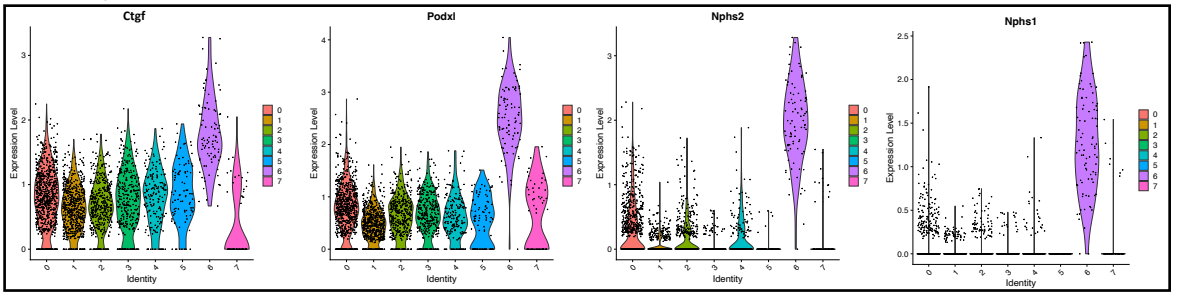

Juxtaglomerular cell

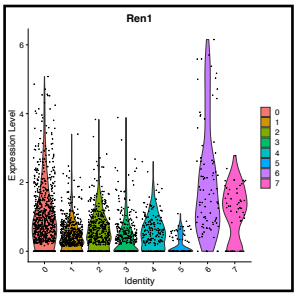

Mesangium

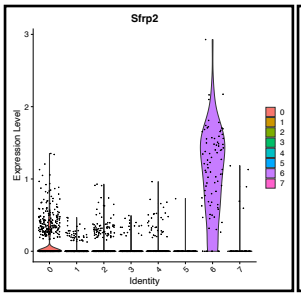

Fibroblast

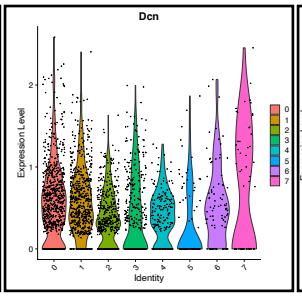

VSMC

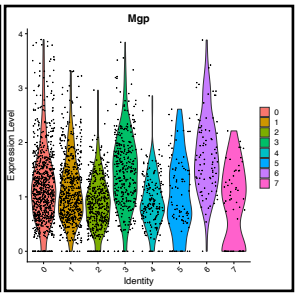

Collecting duct

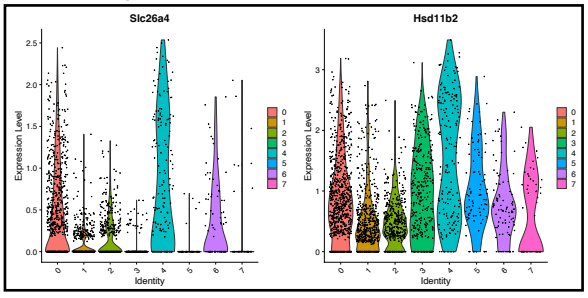

Supplementary Figure 8

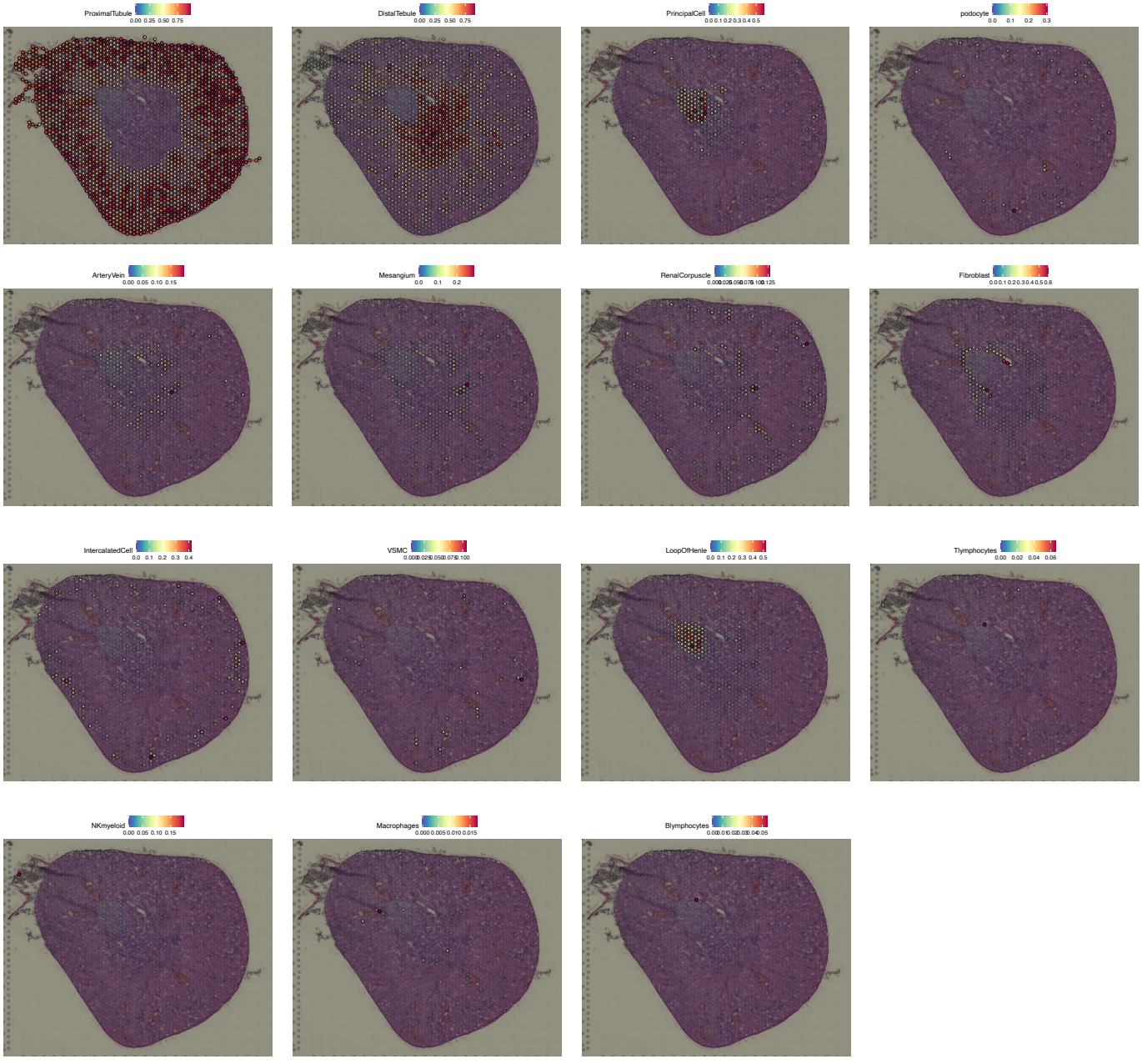

Supplementary Figure 9

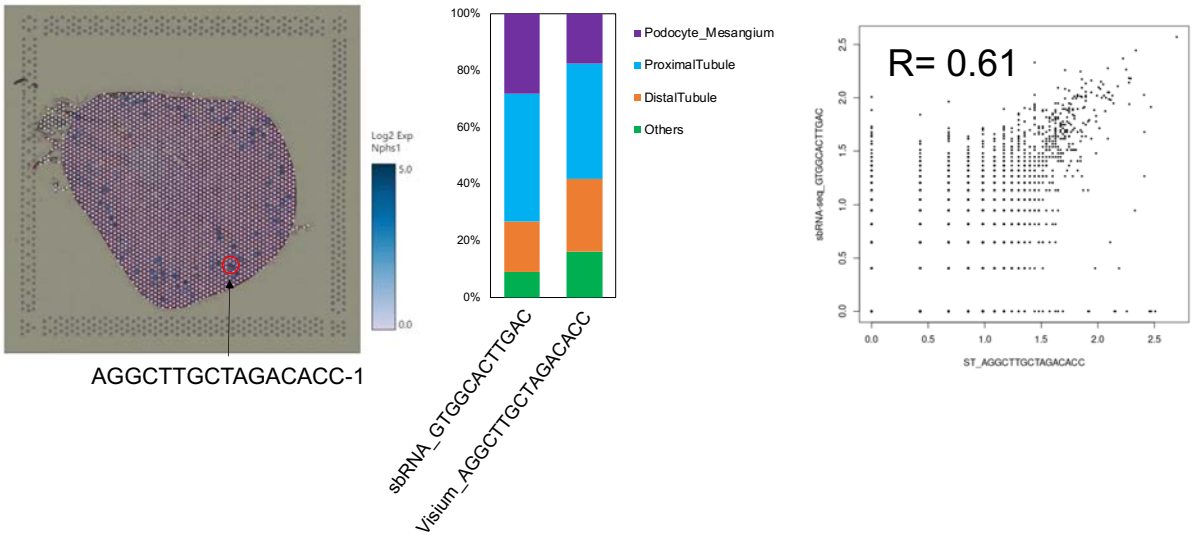

Supplementary Figure 10

a

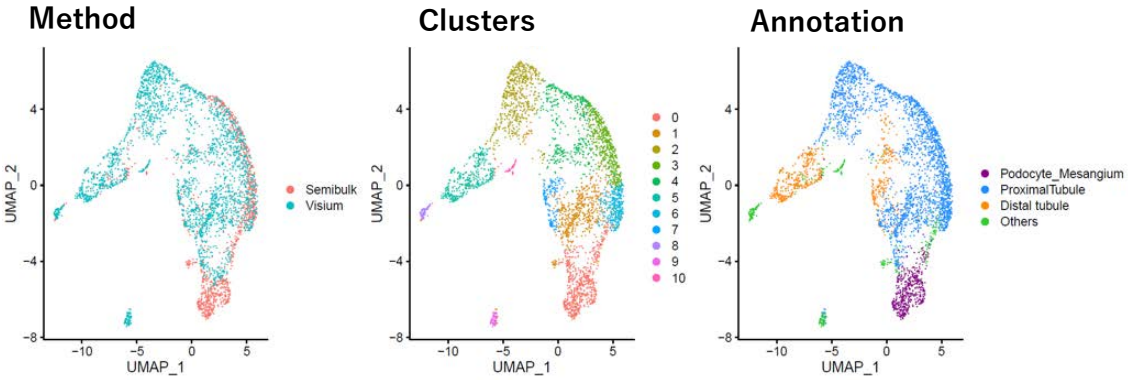

b

Cluster	Semibulk		Visium		Annotation
0	495	43%	135	5%	Podocyte and Mesangium
1	83	7%	477	19%	Proximal Tubule
2	20	2%	506	20%	Proximal Tubule
3	231	20%	257	10%	Proximal Tubule
4	70	6%	341	14%	Proximal Tubule
5	33	3%	333	13%	Distal tubule
6	181	16%	153	6%	Proximal Tubule
7	26	2%	123	5%	Distal Tubule
8	3	0%	63	3%	Others
9	2	0%	62	3%	Others
10	14	1%	27	1%	Others
Total	1158		2477		

c

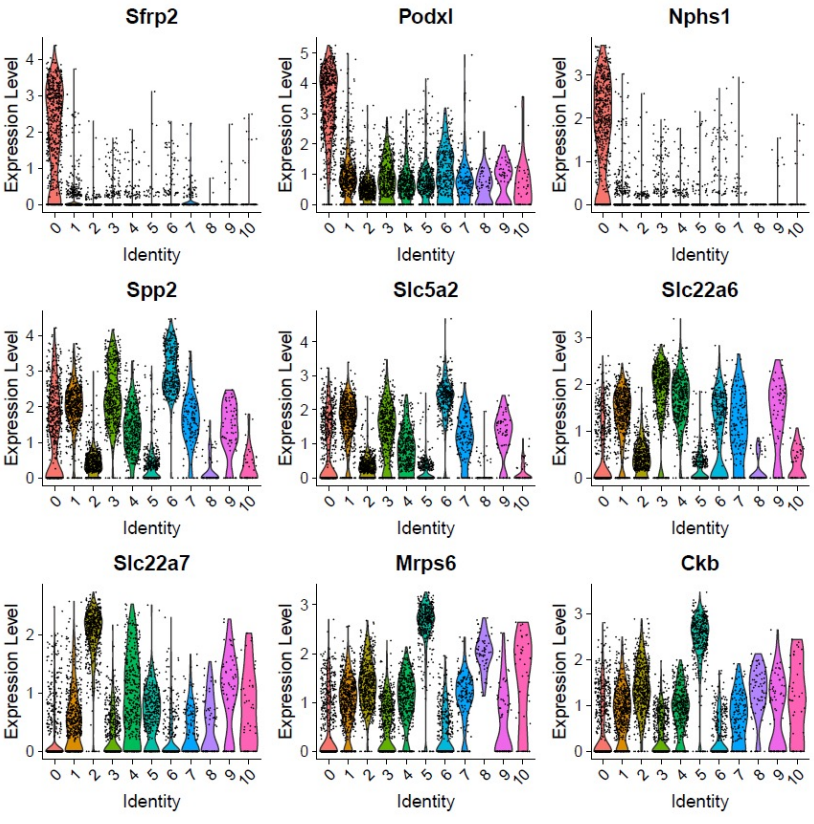

Supplementary Figure 11

a

Case A

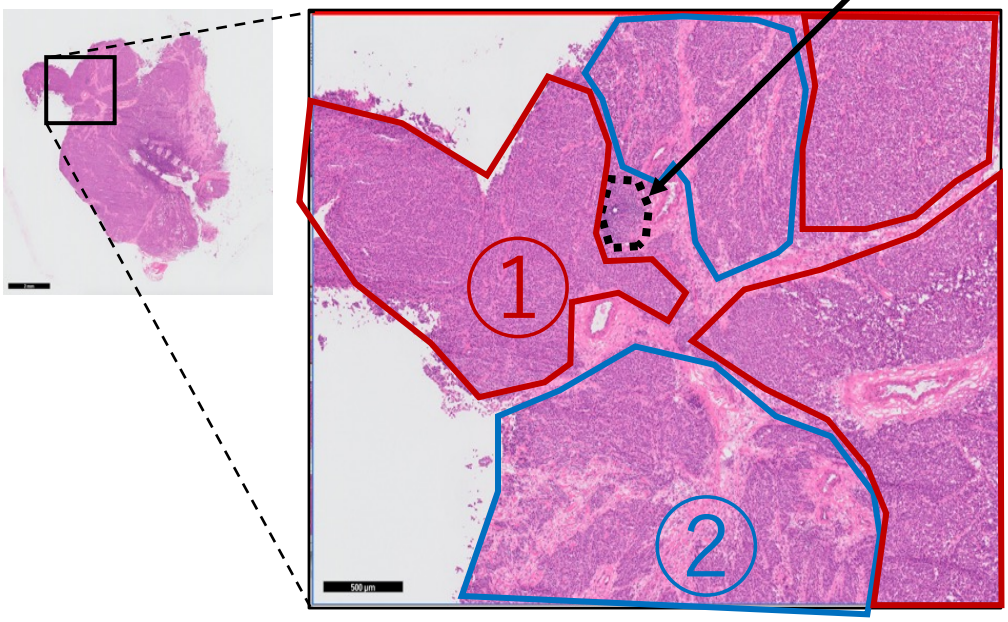

① Invasive carcinoma area showing a substantial and solid tumor growth pattern.

②Invasive carcinoma areas showing a strong stromal reaction growth pattern. There are many solitary areas with loose tumor connectivity.

b

Case B

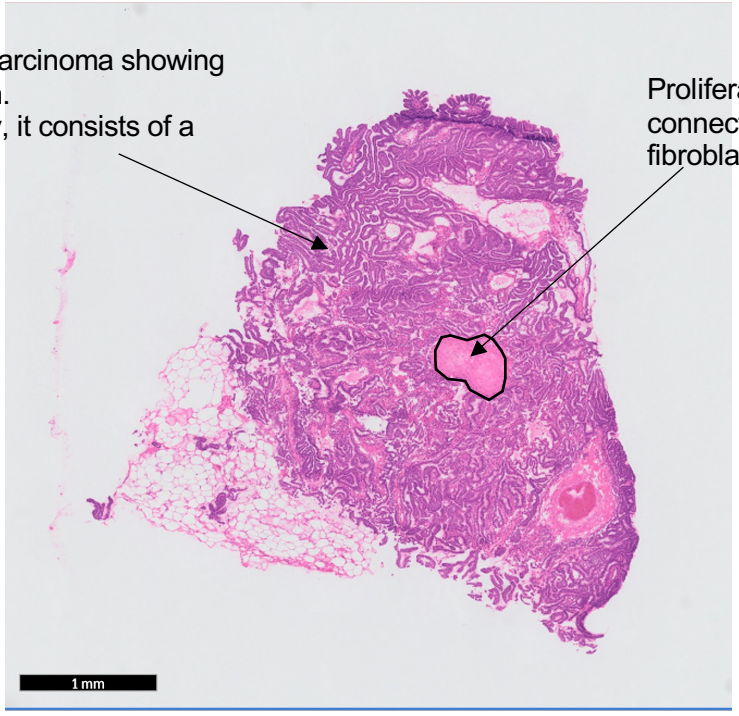

Supplementary Figure 12

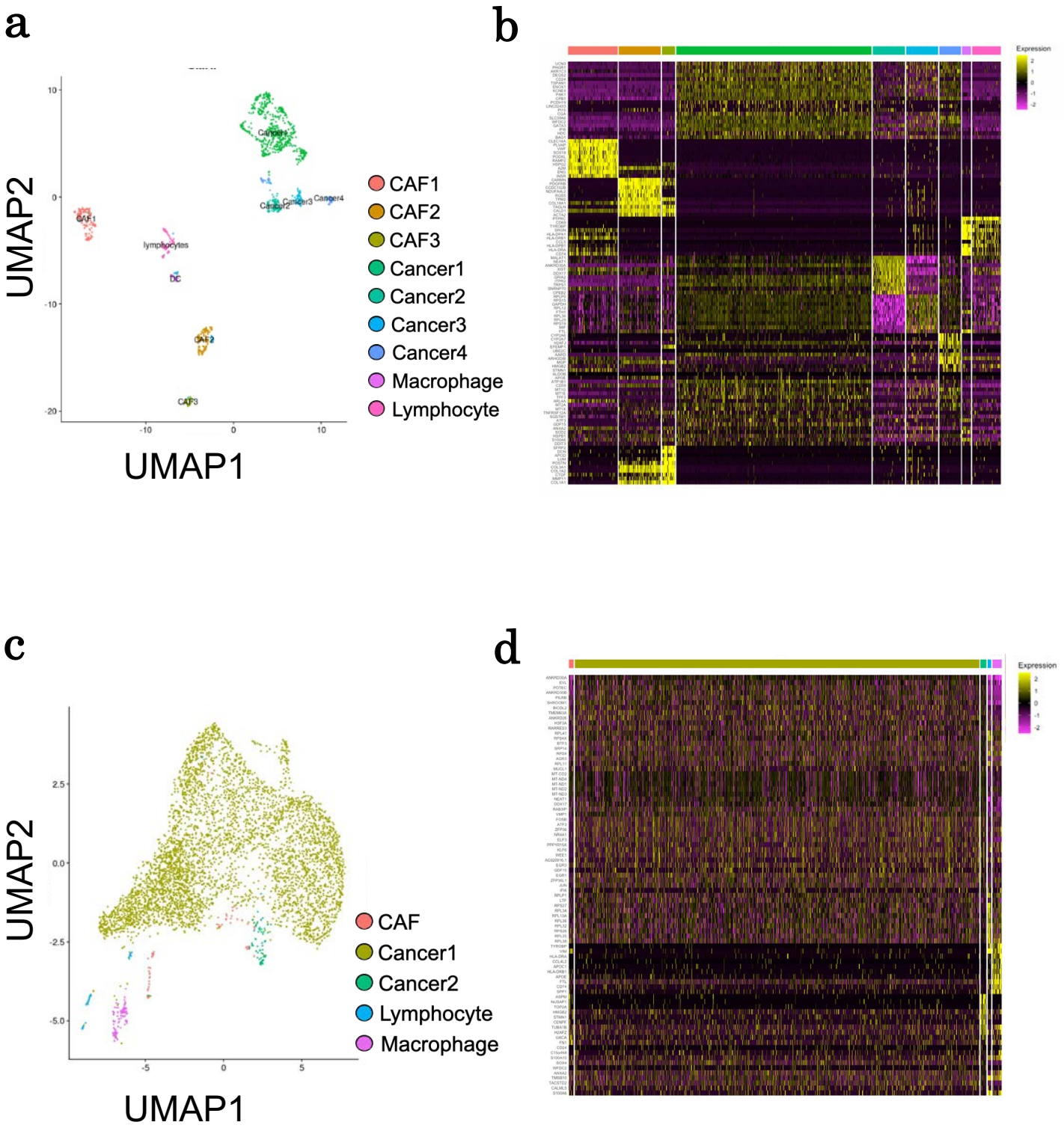

Supplementary Figure 13

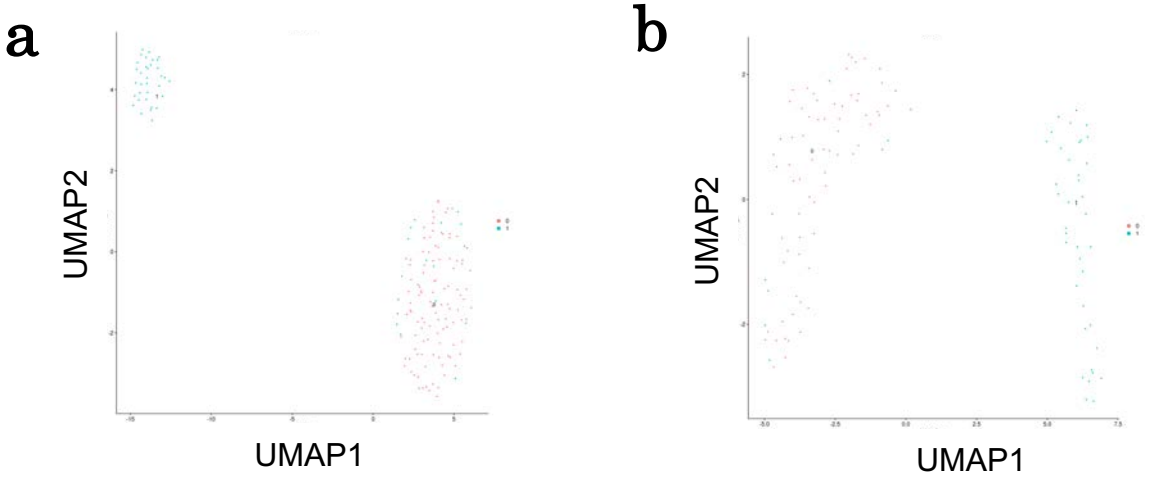

Supplementary Figure 14

a

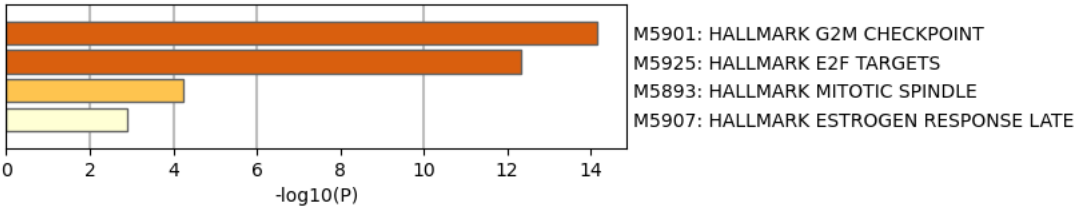

b

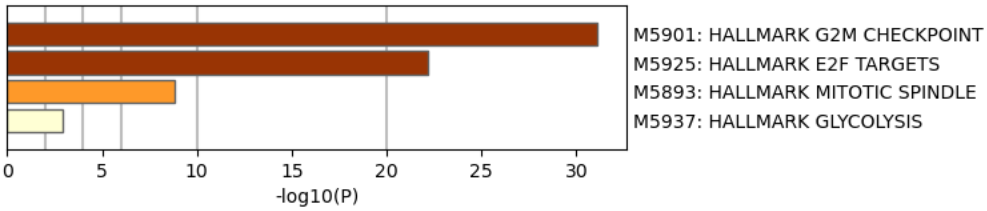

Supplementary Figure 15

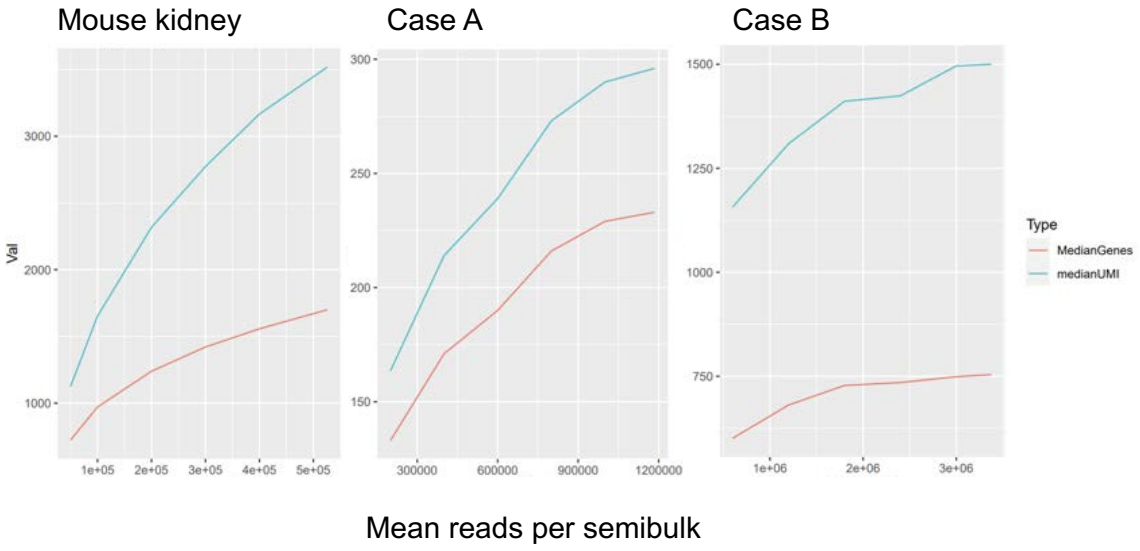

Supplementary Figure 16

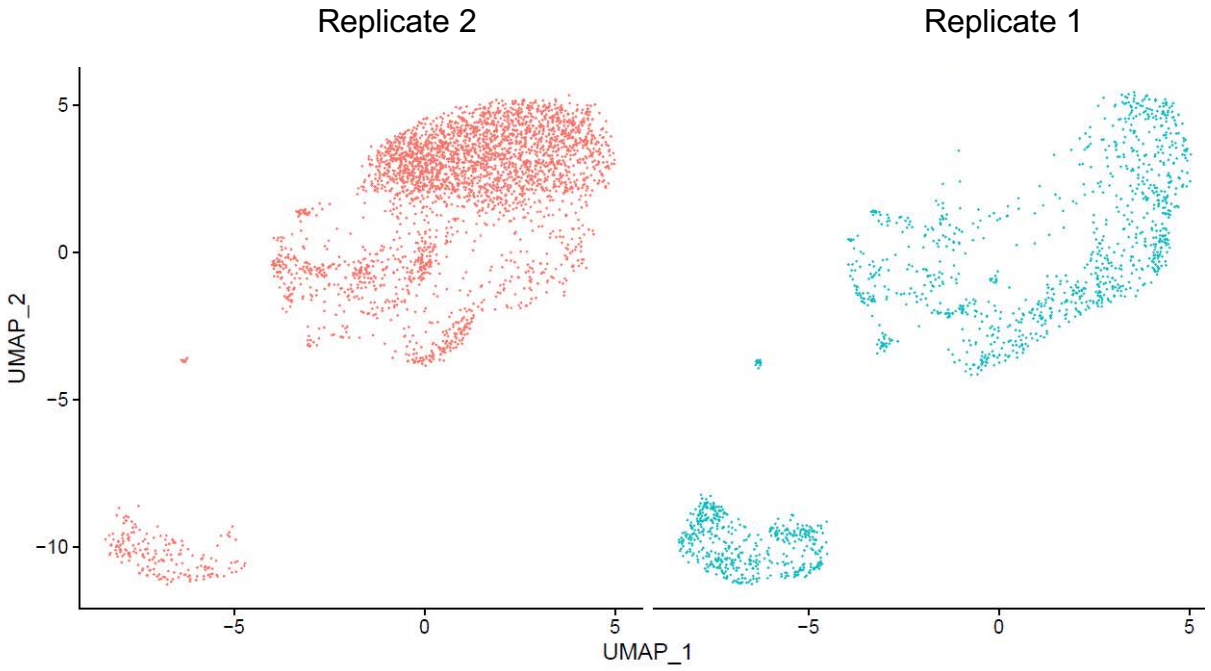

Supplementary Figure 17
