# Supplementary material for: Semibulk RNA-seq analysis as a convenient method for measuring gene expression statuses in a local cellular environment
Source: Sci Rep. 2022 Sep 12;12:15309. doi: 10.1038/s41598-022-19391-2 (PMC9468030; doi:10.1038/s41598-022-19391-2)
Supplement: Supplementary file 2 — Supplementary Information. [file 41598_2022_19391_MOESM2_ESM.pdf]

LISTS OF SUPPLEMENTARY FIGURE AND TABLES

Supplementary Figure 1. Evaluation of cell viability in semibulks

Semibulks derived from mouse kidney was stained using 0.2% trypan blue (final concentration). The scale bars represent 200 μm. We consider that this sort of mechanical dissociation should be less damaging as it does not include any incubation time, which may allow RNA degradation. In addition, since cryopreservation causes cellular damage, it may result in RNA degradation. To evaluate the cellular damage induced by mechanical dissociation, we performed trypan blue staining of semibulks prepared from murine kidney. Except for small portion of cells and tissue gap, almost no cells were stained, showing that they are alive.

Supplementary Figure 2. Microfluidics of the 10x Chromium and custom PDMS device for sbRNA-seq

Microfluidics of Chromium Next GEM G chip (10x Genomics) for scRNA-seq (**a**) and custom PDMS device for sbRNA-seq (**b**). The flow directions of cells or semibulks and beads is indicated by arrows. Scale bars represent 100 μm (**a**) and 1 mm (**b**).

Supplementary Figure 3. Expression patterns of cell-type marker genes in sbRNA-seq data of mouse kidney

Violin plots of expression levels for each cell-type marker in mouse kidney sbRNA-seq. Marker genes are shown as below; proximal tubule, S1: *Spp2*, *Slc5a2*[27]; proximal tubule, S2: *Slc22a6*[50]; proximal tubule, S3: *Slc22a7*[27]; Distal tubule: *Egf*, *Slc12a1*, *Tmem52b*, *Umod*[27,51]; podocyte: *Ctgf* (*Ccn2*), *Podxl*, *Nphs1*, *Nphs2*[27-29]; juxtaglomerular cell: *Ren1*[52]; mesangium: *Sfrp2*[29]; fibroblast: *Dcn*[27]; vascular smooth muscle cells (VSMC): *Mgp*[53]; collecting duct: *Slc26a4, Hsd11b2*[54]. Log-normalized expression levels were calculated by NormalizeData function of Seurat[24] with a scale factor 1000. Violin plots were generated with Seurat's VlnPlot function.

Supplementary Figure 4. Cellular components estimated by Seurat, SPOTlight, and RCTD from semibulk datasets of mouse kidney.

Cell-type proportions of mouse kidney sbRNA-seq inferred from Seurat[24], SPOTlight[22], and RCTD[23]. The upper colored keys show the semibulk classifications manually annotated from cell-type markers in **Figure 3b**. The heatmap of the cellular-type proportions was prepared using pheatmap of R.

For Seurat, cell-type proportions were estimated using the FindTransferAnchors function of Seurat v4.0.1 with default parameters. For SPOTlight,

first, marker genes for each cell-type were detected using the FindAllMarker function of Seurat with the following options: logfc.threshold = 1 and min.pct = 0.8. The cell-type fractions were then estimated using SPOTlight v1.0.1 following the instruction manual with the cell. For RCTD, estimation of cell-type fractions was performed using RCTD of spacexr v2.0.1 following the manual with some modifications. To create a reference object including the entire normalized single cell data, the reference function was used with the following options: require_int = F and n_max_cells = 20000. The option of RCTD for the minimum number of cells required per cell-type was set to 20.

We found that Seurat and RCTD showed similar results with CIBERSORTx (**Fig. 3c** and **Supplementary Fig. 4**). For SPOTlight, T lymphocytes, VSMCs, intercalated cells, principal cells, and cells from the loop of Henle (LoH) were predicted as major components, whereas proximal tubular cells were predicted as relatively minor components in the kidney. Previous studies show that proximal tubular cells were the most abundant cell-type in the mouse kidney[5,6]. Moreover, proximal tubular cells and LoH are present at different locations, the cortex and medulla, respectively[27]. However, these cell types were frequently predicted in the same semibulks in the SPOTlight results. These discrepancies were not observed in the CIBERSORTx, Seurat, or RCTD results. Therefore, at least in our case, CIBERSORTx, Seurat, and RCTD are a more robust tool for deconvolution compared with SPOTlight.

Supplementary Figure 5. Correlation between the gene expression of pseudo-semibulks and real semibulk data.

Boxplot of the Pearson correlation coefficients between real semibulk and corresponding pseudo-semibulk. The expression patterns of virtual semibulk were constructed from the cellular compositions that were predicted by CIBERSORTx[25], Seurat[24], SPOTlight[22], or RCTD[23] and the averaged gene expression of scRNA-seq for each cell-type.

To further validate the precise prediction of the cellular components, we generated the pseudo-semibulk based on the predicted cellular components by CIBERSORTx, Seurat, SPOTlight, and RCTD (**Supplementary Fig. 5**). When we compared the correlation between these pseudo-semibulks and real semibulks, we found that the average of the correlation coefficients was 0.57 in CIBERSORTx, Seurat, and RCTD and 0.46 in SPOTlight. These results again suggest that CIBERSORTx is a reasonable bioinformatics tool, at least for our purpose (**Supplementary Figs. 4** and **5**).

Supplementary Figure 6. Evaluation of cell density

(**a**) Log 2-normalized unique modifier identifiers (UMI) counts of each cluster. (**b**) Log 2-

normalized UMI counts in each spot. Enlarged images of the regions around cluster 1, 5, and 7 are included as well. For cluster 1 and 5, the number of manually counted nuclei is shown below the images.

UMI counts roughly correlates with cell density or mRNA cell content[34]. To roughly estimate cell density at each spot, we compared UMI counts among spots (**Supplementary Fig. 6a**). Although the spots of cluster 7, mainly derived from damaged thin tissue fragments, showed lower UMI counts compared to other clusters, others showed almost comparable UMI counts among each other. Furthermore, we manually counted the number of nuclei by inspecting the HE image. A total of 326 and 422 of nucleus were identified in an ~0.2 mm square region for the regions of clusters 1 and 5, which have relatively high and low UMI counts, respectively (**Supplementary Fig. 6b**). Therefore, we concluded that our Visium planar should have a relatively flat cellular density across the observed region.

Supplementary Figure 7. Clustering analysis of mouse kidney ST data

A heatmap of differentially expressed genes in each cluster shown in **Figure 4a**. Representative cell-type markers are manually represented in the margin.

Supplementary Figure 8. Expression patterns of cell-type marker genes in mouse kidney ST data

Violin plots of expression levels for each cell-type marker in mouse kidney ST. The same marker genes as **Supplementary Figure 3** are shown.

Supplementary Figure 9. Localization of cell types on the tissue section

Localization for each cell-type. To visualize cell-type proportions on the tissue section, we used the SpatialFeaturePlot function of Seurat[24].

Supplementary Figure 10. Comparison of multiple platforms, sbRNA-seq and ST

Comparison of expression patterns among multiple platforms, sbRNA-seq and ST. The HE image with *Nphs1* expression levels, the cell fraction for the semibulk and the Visium spot, and the scattering plot of gene expression between them are shown. The Pearson correlation coefficient is shown in the graph as well.

Supplementary Figure 11. UMAP plot of semibulk RNA-seq and Visium ST.

(**a**) UMAP plot created following the integration procedure of Seurat. Plot colored by method (left), clusters (middle), and classifications manually annotated by cell-type

markers in **Figure 3b** and **Supplementary Figure 5** (right) are shown, respectively. (**b**) Breakdown of the number of semibulks or spots is shown. The annotation shows the most frequently observed cell-type in each cluster. (**c**) Violin plots of the expression levels for each cell-type marker in mouse kidney semibulks and ST. Marker genes are shown as follows: proximal tubule (S1), *Spp2* and *Slc5a2*[27]; proximal tubule (S2), *Slc22a6*[50]; proximal tubule (S3), *Slc22a7*[27]; distal tubule in outer medulla, *Mrps6* and *Ckb*[27]; podocyte, *Podxl* and *Nphs1*[27,29]; mesangium, *Sfrp2*[29].

We attempted to visualize the semibulk and Visium datasets in the same UMAP plot. After reducing the influence derived from the difference in the methodologies by employing the integration procedure of Seurat, we plotted the respective datasets on the same planar. The data showed a general overlap. Nevertheless, analyzing in more detail, there were several places which did not show an exact overlap. For example, the semibulk data showed a higher rate of data points for glomerular cells (Cluster 0) and S1 proximal tubules (Cluster 6) and a lower rate of data points for S3 proximal tubules (Cluster 2) and subset of distal tubules (Cluster 5) compared with the Visium data. Glomerular cells and S1 proximal tubules are located in the cortex[27]. On the other hand, S3 proximal tubules and the subset of distal tubules are located in the outer medulla[27]. All indicated a higher representation for the medulla on the Visium side.

Supplementary Figure 12. Histological images of human clinical specimens.
Hematoxylin and eosin staining of Case A (**a**) and B (**b**), with detailed annotations. Case A is derived from the same specimens as a tumor tissue of Case 8 used in our previous study[45].

Supplementary Figure 13. Analysis of scRNA-seq reference of breast cancer specimens
(**a**) UMAP plot of the scRNA-seq reference dataset in Case A. scRNA-seq data indicated roughly four clusters of cancer cells (Cancer1–4) and three clusters for cancer-associated fibroblast cells (CAF1–3), macrophage groups, and lymphocytes. (**b**) A heatmap representing differential expressed genes of each cell-type in Case A. (**c**) UMAP plot of scRNA-seq in Case B. (**d**) A heatmap represents differentially expressed genes of each cell-type in Case B.

Supplementary Figure 14. Analysis of sbRNA-seq of breast cancer specimens
(**a**) UMAP plot of sbRNA-seq in Case A. (**b**) UMAP plot of sbRNA-seq in Case B.

Supplementary Figure 15. Gene set enrichment analysis

(a) MSigDB v5.1 Hallmark gene set collection (HALLMARK) pathway analyses were conducted using the selected differentially expressed genes (the top 30 upregulated genes in Cancer4 subgroup of Case A) by Metascape[42]. Each band represents each of the enriched terms or pathways colored according to the −log 10 p-value. **(b)** HALLMARK pathway analyses using the selected differentially expressed genes (the top 30 upregulated genes in Cancer2 subgroup of Case B) by Metascape.

Supplementary Figure 16. Saturation curve of semibulk RNA-seq.

The plots for the saturation curve of sbRNA-seq for mouse kidney and breast cancer specimens (Cases A and B). The reads of sbRNA-seq were randomly sampled, and the median number of genes and UMI per semibulk was estimated.

For clinical samples, because the quality was not always high, the number of UMIs and genes was not always high (**Supplementary Fig. 16** and **Supplementary Table 5**), which also occurred when the commercial single cell platform is used. However, for the mouse kidney, the median number of detected genes per semibulk was 1,700, and the median UMIs per semibulk were 3,500 (**Supplementary Table 4**), which is compatible with the common single cell datasets. For example, in public scRNA-seq datasets for human peripheral blood mononuclear cells released by 10x Genomics (v3.1 Chemistry), the median number of genes per cell was 1,800–2,200, and the median UMI counts per cell were 5,700–7,700, respectively[55,56]. Therefore, we believe that the numbers for sbRNA-seq should not be surprising at a given sequencing depth. Moreover, note that the sequencing depth of the semibulks datasets was not saturated in the mouse kidney data (**Supplementary Fig. 16**). Therefore, we expect that these numbers could be somewhat improved by additional sequencing.

Supplementary Figure 17. UMAP analysis of experimental replicates

UMAP plot of kidney sbRNA-seq data after additional sequencing and the new experimental replicate generated by Seurat with default settings. The replicates were clustered together, showing minimum if any batch effects between replicates.

REFERENCES

50. Hwang, J. S., Park, E. Y., Kim, W. Y., Yang, C. W. & Kim, J. Expression of OAT1 and OAT3 in differentiating proximal tubules of the mouse kidney. *Histol. Histopathol.* **25**, 33–44 (2010).
51. Bais, A. S. *et al.* Single-cell RNA sequencing reveals differential cell cycle activity in key cell populations during nephrogenesis. *Sci. Rep.* **11**, 22434 (2021).
52. Clark, A. F. *et al.* Renin-1 is essential for normal renal juxtaglomerular cell granulation and macula densa morphology. *J. Biol. Chem.* **272**, 18185–18190 (1997).
53. Bjørklund, G. *et al.* The Role of Matrix Gla Protein (MGP) in Vascular Calcification. *Curr. Med. Chem.* **27**, 1647–1660 (2020).
54. Chen, L. *et al.* Transcriptomes of major renal collecting duct cell types in mouse identified by single-cell RNA-seq. *Proc. Natl. Acad. Sci. U. S. A.* **114**, E9989–E9998 (2017).
55. 5k Peripheral Blood Mononuclear Cells (PBMCs) from a Healthy Donor with a Panel of TotalSeq™-B Antibodies (Next GEM) - 10x Genomics. Available at: https://www.10xgenomics.com/resources/datasets/5-k-peripheral-blood-mononuclear-cells-pbm-cs-from-a-healthy-donor-with-cell-surface-proteins-next-gem-3-1-standard-3-0-2. (Accessed: 10th August 2022)
56. 5k Peripheral Blood Mononuclear Cells (PBMCs) from a Healthy Donor (Next GEM) - 10x Genomics. Available at: https://www.10xgenomics.com/resources/datasets/5-k-peripheral-blood-mononuclear-cells-pbm-cs-from-a-healthy-donor-next-gem-3-1-standard-3-0-2. (Accessed: 10th August 2022)

Supplementary Table 1. Summary of the mixture of mouse and human cell lines

	Human	Mouse	Multiplet
Total number of reads		561,042,047	
Number of reads after filtering by UMI-tools		380,478,653	
Number of reads uniquely mapped to human		161,764,024	
Number of reads uniquely mapped to mouse		78,403,053	
% uniquely mapped reads		63.1	
Total cells		3,051	
Mean read per cell		183,888	
Number of cells (% in total cells)	1,451 (47.6%)	1,416 (46.4%)	184 (6.0%)
Total UMI counts	3,706,423	2,677,476	496,043
Median UMI counts per semibulk	2,018	1,592	2,150
Median genes per cell	1,016	773	
% mitochondrial UMI counts	2.9	0.0	

Supplementary Table 2. General statistics of Visium spatial transcriptome data of mouse kidney

	Mouse kidney
Number of spots	2,477
Number of reads	575,661,696
Mean reads per spot	232,403
Median UMI counts per spot	27,937
Median genes per spot	6,200
Total genes detected	20,620
Reads mapped to genome (%)	95.1
Sequencing saturation (%)	83.1

Supplementary Table 3. General statistics of the scRNA-seq reference of breast cancer specimens

	Case A	Case B
Number of reads	353,015,615	347,134,191
Number of cells	2,181	8,294
Mean reads per cell	161,859	41,853
Median UMI counts per cell	7,113	2,535
Median genes per cell	1,868	1,064
Total genes detected	23,385	22,994
Reads mapped to genome (%)	90.2	89.4
Sequencing saturation (%)	68.9	28.8

Supplementary Table 4. Summary of data after increasing sequence depth and an experimental replicate for semibulk RNA-seq data

	With increased sequencing depth	Experimental replicate
Total number of reads	785,852,543	1,066,532,345
Number of reads after filtering by UMI-tools	191,216,992	716,233,293
Number of uniquely mapped reads	121,148,023	349,383,978
% uniquely mapped reads	65.6	49.1
Number of semibulks	1,495	3,205
Mean reads per semibulk	525,654	332,771
Total UMI counts	8,201,489	10,872,572
Median UMI counts per semibulk	3,518	2,197
Median genes per semibulk	1,700	1,196
% mitochondrial UMI counts	0.2	2.5

In the semibulks, cells would stick together with strong centrifugation. Therefore, it was inherently difficult to concentrate the semibulk suspension to the optimal concentration for efficient droplet encapsulation in the original protocol. To address this issue, we firstly captured the semibulks on a 40 µm cell strainer. After letting the extra buffer flow through, the semibulks were released by inverting the strainer and placing a minimum amount of buffer from the other side. Through this simplified procedure, we could enrich the semibulk suspension to >4 semibulks/µl.

Supplementary Table 5. General statistics of sbRNA-seq of breast cancer specimens after increasing sequence depth

	Case A	Case B
Total number of reads	672,922,174	671,573,377
Number of reads after filtering by UMI-tools	306,684,688	204,746,161
Number of uniquely mapped reads	163,097,458	137,325,460
% uniquely mapped reads	53.7	67.3
Number of semibulks	568	199
Mean reads per semibulk	1,184,722	3,374,741
Total UMI counts	304,473	468,493
Median UMI counts per semibulk	296	1,500
Median genes per semibulk	233.5	754
% mitochondrial UMI counts	4.5	3.6

Supplementary Table 6. Comparison with other platforms

	Bulk RNA-seq	scRNA-seq	Visium	sbRNA-seq
Throughput	Low (≤100 samples)	High (≤10,000 cells)	High (≤4,992 spots)	High (≤3,200 semibulks)
Technical difficulty	Low	Middle	High	Middle
Spatial information	Too low resolution	not preserved	preserved	preserved
